# CT-based multi-station lymph-node radiomics for preoperative prediction of lymph node metastasis in colorectal cancer: a proof-of-concept comparison with primary-tumor radiomics

**DOI:** 10.1186/s41747-026-00814-y

**Published:** 2026-10-12

**Authors:** Zejin Lin, Jue Wang, Hairong Chen, Qun Bao, Ming Xu, Shang Yang, Hanxiao Zhang, Guang-Zhong Yang, Peng Sun

**Affiliations:** 1 Department of General Surgery, Tongren Hospital, Shanghai Jiao Tong University School of Medicine, Shanghai, China; 2 Shanghai Key Laboratory of Flexible Medical Robotics, Tongren Hospital, Institute of Medical Robotics, Shanghai Jiao Tong University, Shanghai, China; 3 Institute of Medical Robotics, School of Biomedical Engineering, Shanghai Jiao Tong University, Shanghai, China; 4 Department of Imaging, Tongren Hospital, Shanghai Jiao Tong University School of Medicine, Shanghai, China; 5 Bengbu Medical University, Bengbu, China

**Keywords:** Colorectal neoplasms, Lymphatic metastasis, Radiomics, Risk assessment, Tomography (x-ray computed)

## Abstract

**Objective:**

Accurate preoperative prediction of lymph node metastasis (LNM) in colorectal cancer remains challenging using conventional CT assessment. This proof-of-concept study compared CT-based multi-station lymph-node radiomics with primary-tumor radiomics for patient-level preoperative prediction of LNM.

**Materials and methods:**

This single-center retrospective study included patients with pathologically confirmed colorectal cancer who underwent preoperative contrast-enhanced CT and surgical resection. Patients were divided into training and validation cohorts. Radiomics features were extracted from the primary tumor and visible regional lymph nodes across multiple nodal stations. Three radiomics strategies were developed and compared: primary-tumor radiomics, multi-station lymph-node radiomics, and combined radiomics. Model performance was assessed using the area under the receiver operating characteristic curve (AUC), calibration analysis, decision curve analysis, and five-fold cross-validation. Pairwise AUCs were compared using the DeLong test. A clinical-radiomic model was further constructed by integrating the optimal radiomics strategy with preoperative clinical variables.

**Results:**

In the validation cohort, the multi-station lymph-node radiomics model outperformed the primary-tumor radiomics model for predicting LNM (AUC, 0.864 *versus* 0.637; *p* = 0.0055). The combined radiomics model achieved an AUC of 0.840, without significant improvement over the lymph-node model (*p* = 0.2211). After integration with clinical variables, the clinical-radiomic model achieved an AUC of 0.879.

**Conclusion:**

CT-based multi-station lymph-node radiomics showed significantly higher validation performance than primary-tumor radiomics for patient-level preoperative LNM prediction in colorectal cancer. However, this single-center proof-of-concept model is not ready for direct clinical implementation and requires multicenter external validation before clinical translation.

**Key Points:**

***Question*** Can CT-based multi-station lymph-node radiomics improve preoperative nodal metastasis prediction beyond primary-tumor radiomics in patients with surgically treated colorectal cancer?

***Findings*** Multi-station lymph-node radiomics achieved higher validation performance than primary-tumor radiomics for patient-level prediction of pathological lymph-node metastasis in colorectal cancer.

***Relevance statement*** CT-based multi-station lymph-node radiomics may support individualized preoperative nodal risk stratification in colorectal cancer, but multicenter validation and prospective evaluation are required before clinical translation.

**Graphical Abstract:**

**Figure:**
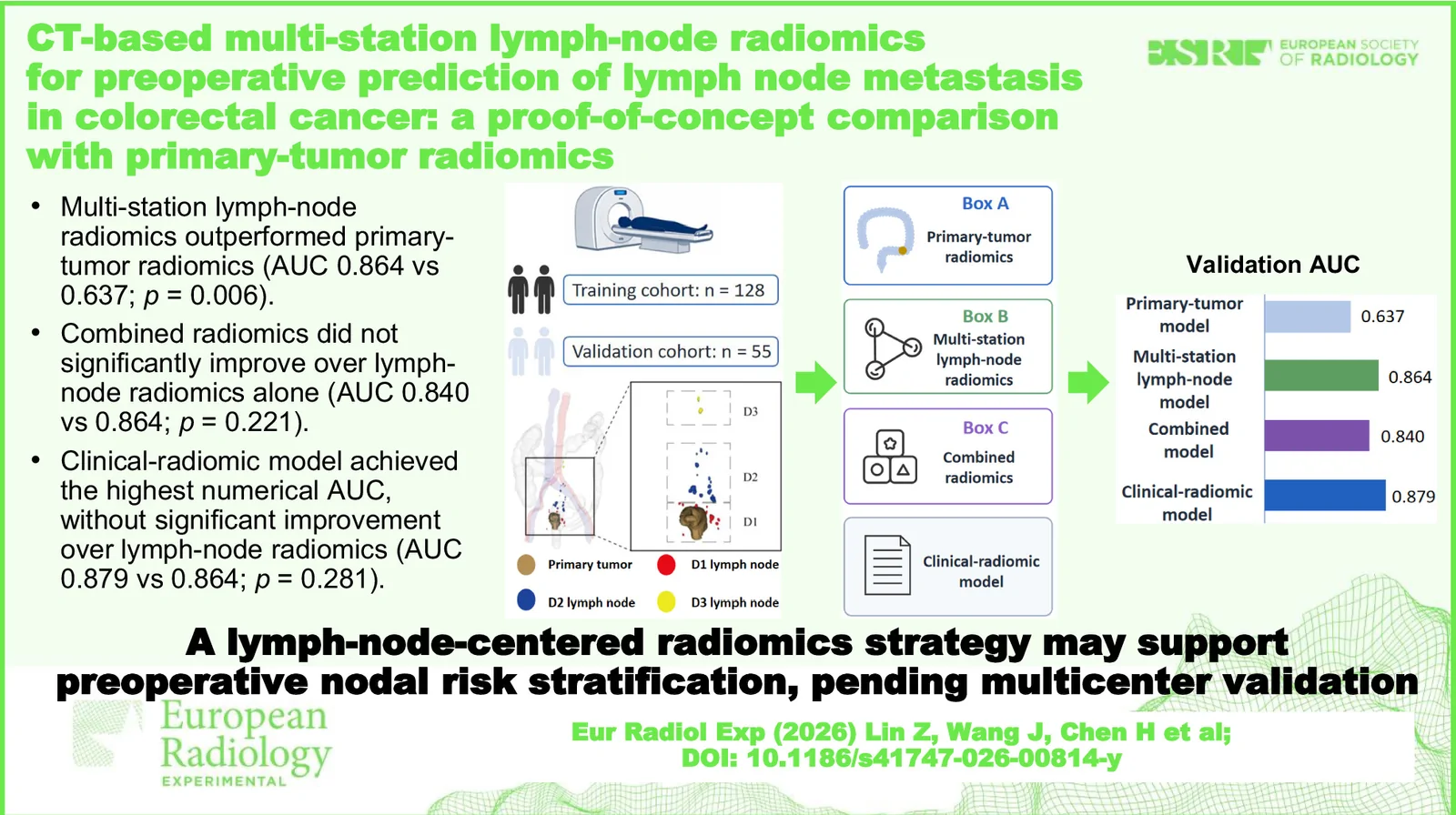

## Background

Globally, colorectal cancer (CRC) ranks among the most frequently diagnosed malignancies and is a major contributor to cancer-related mortality [1–3]. Lymph node metastasis (LNM) is a major route of tumor dissemination in CRC and a key determinant of postoperative recurrence and survival [4–6]. In addition, lymph node status strongly influences treatment decision-making, including the selection of neoadjuvant therapy, surgical planning, and postoperative adjuvant treatment [7–9]. Accordingly, accurate determination of lymph node status before surgery is of great importance for tailoring individualized treatment strategies. However, conventional imaging assessment still relies largely on morphologic criteria, such as lymph node size and shape, which remain insufficiently accurate for the reliable identification of metastatic lymph nodes.

Radiomics has emerged as a promising noninvasive approach for extracting quantitative imaging information beyond visual assessment from routine medical images [10, 11]. Radiomics has shown potential in CRC for characterizing tumors, stratifying risk, and predicting LNM before surgery [12]. Nevertheless, in the majority of previous studies, lymph node status was predicted indirectly on the basis of radiomic features derived from the primary tumor [12, 13]. For example, Huang et al previously reported a CT-based radiomics nomogram integrating portal venous phase tumor features and clinical variables for preoperative LNM prediction in CRC [14]. Subsequent studies also explored clinical-radiomics models based predominantly on primary-lesion features [13, 15].

From a biological and translational standpoint, direct radiomic characterization of regional lymph nodes may provide more relevant information for patient-level LNM risk stratification than conventional primary-tumor-centered approaches. Indeed, lymph-node-focused radiomics has shown promise in rectal cancer, although existing studies remain limited and heterogeneous in imaging modality, target-node definition, and model design [16]. In particular, radiomics studies based on direct analysis of lymph nodes have focused on isolated nodal targets or selected nodal regions, whereas the potential value of multi-station lymph-node characterization remains insufficiently explored in CRC. It also remains unclear whether multi-station lymph-node radiomics provides incremental or superior predictive information compared with conventional primary-tumor radiomics for patient-level preoperative prediction of LNM.

Therefore, this proof-of-concept study aimed to develop and compare three CT-based radiomics strategies for patient-level preoperative prediction of LNM in CRC: primary-tumor radiomics, multi-station lymph-node radiomics, and combined radiomics integrating both feature sets. Based on the optimal radiomics strategy, we further developed a clinical-radiomic model to evaluate the incremental value of preoperative clinical variables and the potential clinical usefulness of this lymph-node-centered radiomics approach.

## Materials and methods

### Study population

**Fig. 1:**
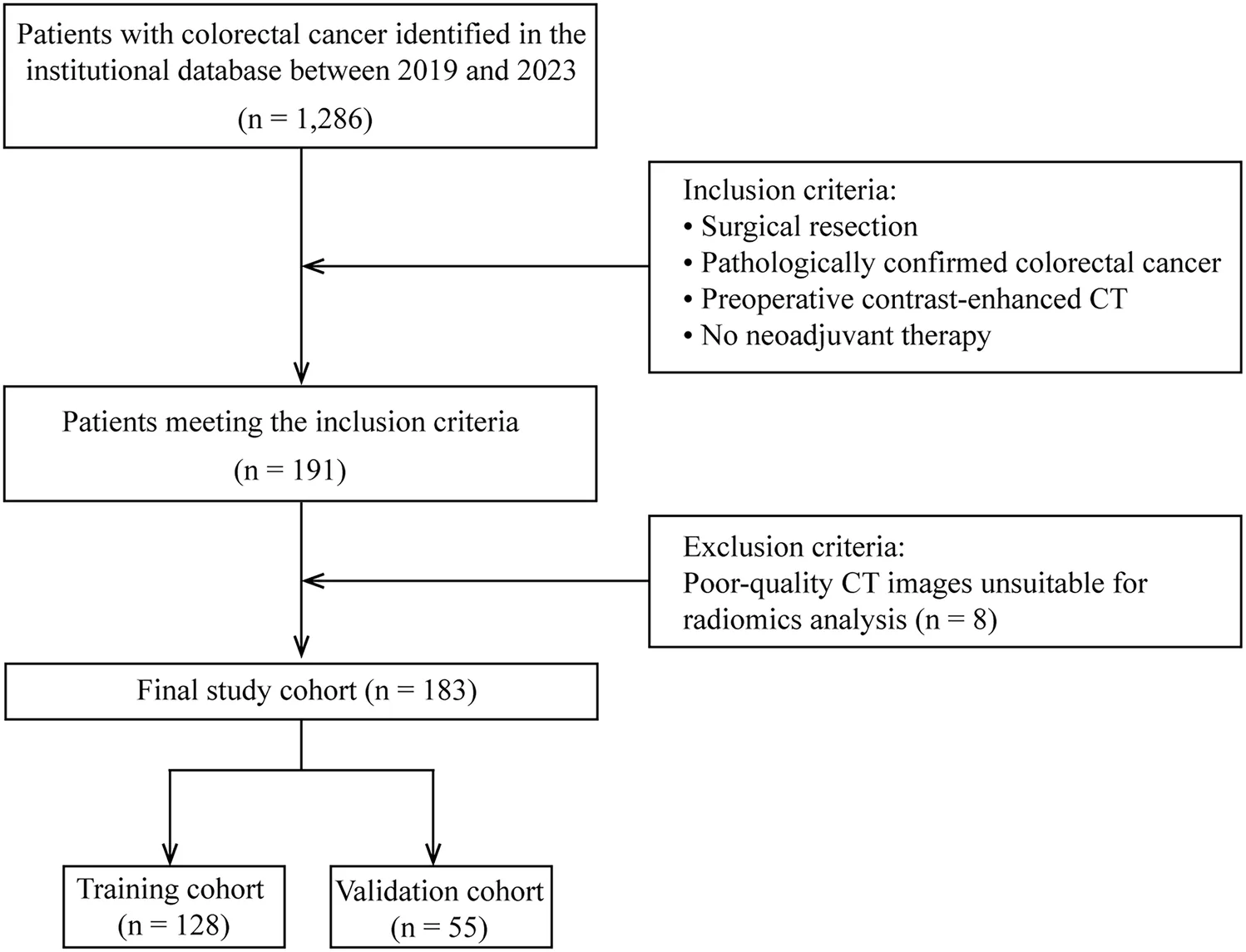
Flowchart of patient selection

This retrospective study included patients with pathologically confirmed CRC who underwent preoperative contrast-enhanced computed tomography (CT) at Tongren Hospital, Shanghai Jiao Tong University School of Medicine, from 2019 to 2023. The inclusion and exclusion workflow is shown in Fig. 1. Eligible patients were randomly assigned to the training and validation cohorts at a 7:3 ratio using stratified random sampling according to pathological lymph-node metastasis status, with a random seed of 2026. Preoperative clinical information was collected from the electronic medical records. This retrospective study was approved by the institutional review board of the participating institution—Tongren Hospital, Shanghai Jiao Tong University School of Medicine (approval no. K2024-069-01; approval date: November 5, 2024), and the requirement for informed consent was waived.

### CT image acquisition

Preoperative contrast-enhanced abdominopelvic CT was available for all enrolled patients. Imaging was performed on multidetector CT platforms, predominantly LightSpeed Pro 32 (GE Medical Systems, Milwaukee, WI, USA) and Aquilion ONE (Toshiba Medical Systems, Otawara, Japan). For image analysis, arterial-phase thin-section contrast-enhanced CT images were used for delineation of the primary tumor and regional lymph nodes, because this sequence was consistently available across the included patients and provided high spatial resolution for small-node segmentation. The routine acquisition settings generally included a tube voltage of 120 kVp, tube current of 350 mA, thin-slice reconstruction (typically 1.0–1.25 mm), reconstruction interval (1.0–1.25 mm) and a matrix size of 512 × 512. Original images were exported in DICOM format and converted to NIfTI format when needed for subsequent segmentation and radiomics analysis. Because CT examinations were acquired in routine clinical practice using different scanners, subsequent image preprocessing was performed to reduce acquisition-related variability.

### Image quality assessment

Image quality was assessed before segmentation by two investigators experienced in abdominal CT interpretation and radiomics segmentation, who were blinded to pathological lymph-node status. CT examinations were excluded when severe motion artifacts, incomplete coverage of the tumor or regional lymphatic drainage area, marked metal artifacts, or inadequate contrast enhancement precluded reliable delineation of the primary tumor or regional lymph nodes. Disagreements were resolved by consensus after joint review.

### Image segmentation

The primary tumor and regional lymph nodes were segmented on contrast-enhanced thin-section CT images. The regions of interest (ROIs) consisted of the primary lesion and regional lymph nodes from three nodal stations. Both primary-tumor and lymph-node segmentations were performed using a segment anything model (SAM)-assisted interactive workflow followed by manual correction and expert review [17]. SAM was used as an interactive assistance tool to generate initial ROI contours rather than as a fully automated segmentation method. All masks were manually reviewed and corrected when necessary by trained readers, particularly for small lymph nodes or indistinct boundaries, followed by expert confirmation. Annotation time and the proportion of manual correction were not prospectively recorded. All ROIs were contoured on a slice-by-slice basis following lesion boundaries, while avoiding adjacent air, vessels, fat, and normal tissues.

According to the Japanese Society for Cancer of the Colon and Rectum (JSCCR) classification [18], regional lymph nodes were anatomically divided into three nodal stations: D1, pericolic or perirectal lymph nodes adjacent to the primary lesion; D2, intermediate lymph nodes along the main feeding vessels; and D3, principal lymph nodes located at the root of the feeding vessels. Visible regional lymph nodes across these three stations were identified on CT images, and a short-axis diameter of at least 3 mm was used as the lower threshold for annotation (Supplementary Fig. 1).

ROI segmentation was performed by one operator (Z.L., a doctoral student with 5 years of clinical experience) using ITK-SNAP (version 4.4.0) [19], and the delineations were subsequently reviewed and confirmed by a senior expert (M.X., with more than 20 years of experience in CRC). Both observers were blinded to the postoperative pathological lymph-node status during segmentation and review.

### Reference standard

Postoperative pathological lymph-node status was used as the reference standard. The prediction task was defined at the patient level, namely the presence or absence of lymph-node metastasis in each patient, rather than node-by-node classification. Because individual lymph nodes identified on preoperative CT could not be reliably matched to specific pathological lymph nodes after surgical resection, direct node-by-node pathological correlation was not performed.

### Conventional CT-based nodal assessment

To compare the proposed radiomics model with routine radiologic assessment, preoperative CT-based clinical nodal status was extracted from the clinical records. Patients with cN0 were classified as conventional CT-negative, whereas patients with cN1 or cN2 were classified as conventional CT-positive. The diagnostic performance of this conventional CT-based nodal assessment was evaluated against postoperative pathological lymph-node metastasis status at the patient level.

### Radiomics feature extraction and selection

Radiomics features were generated from the segmented ROIs with the PyRadiomics package [20] (version 3.1.0). Before feature extraction, image intensities were normalized at the image level by subtracting the whole-image mean intensity and dividing by the whole-image standard deviation. No intensity clipping was applied during preprocessing. When image-mask geometric mismatch was detected, the mask was resampled to the image space using nearest-neighbor interpolation to preserve label values. Uniform isotropic voxel resampling of all CT images was not performed before feature extraction. Gray-level discretization was performed using a fixed bin width of 25 gray-level units. Original and wavelet-filtered images were used for feature extraction. To ensure comparability among the three radiomics strategies, identical extraction settings were used for the primary-tumor, lymph-node, and combined analyses. The extracted feature set included first-order statistics, shape features, gray-level co-occurrence matrix (GLCM) features, gray-level run-length matrix (GLRLM) features, gray-level size-zone matrix (GLSZM) features, gray-level dependence matrix (GLDM) features, and neighboring gray-tone difference matrix (NGTDM) features.

For lymph-node radiomics, visible regional lymph nodes were assigned to three anatomical nodal stations, namely D1, D2, and D3, according to their location relative to the primary tumor and feeding vessels. Radiomics feature extraction was performed for station-specific lymph-node ROIs rather than a single overall lymph-node mask. For patients with multiple visible lymph nodes within the same station, station-level features were summarized using predefined aggregation statistics, including mean, maximum, median, and standard deviation. Pooled lymph-node features across stations were also generated to capture overall nodal phenotypes at the patient level. Patients with few visible lymph nodes were retained if at least one eligible regional lymph-node ROI could be reliably segmented. Very small lymph nodes below the predefined segmentation threshold or nodes with indistinct boundaries were not included. When a nodal station was absent in a patient, the corresponding features were treated as missing and imputed using median values during preprocessing.

After feature extraction, radiomics features were scaled using *Z*-score normalization. Normalization parameters were estimated in the training cohort and then applied to the validation cohort to avoid information leakage. Feature selection was performed only in the training cohort. Spearman correlation analysis was first applied for preliminary dimensionality reduction, and one feature from each pair with a high correlation coefficient (> 0.9) was removed. Recursive feature elimination (RFE) was then used for further dimensionality reduction, retaining ten candidate features for subsequent selection. Final feature selection was performed using least absolute shrinkage and selection operator (LASSO) regression with 10-fold cross-validation. The full list of selected radiomics features, including original feature names, image filters, feature classes, aggregation statistics, and extraction settings, is provided in Supplementary Table 10.

### Construction of radiomics strategies

Three radiomics strategies were constructed for preoperative prediction of LNM: (a) a primary-tumor radiomics strategy based on features extracted from the primary-tumor ROIs; (b) a lymph-node radiomics strategy using features from the regional lymph-node ROIs; and (c) a combined radiomics strategy integrating both primary-tumor and lymph-node radiomics features. For the combined radiomics strategy, the selected feature sets from the primary-tumor and lymph-node analyses were merged according to patient ID to generate a unified feature matrix. The lymph-node radiomics strategy was specifically designed to directly characterize regional lymph-node phenotypes across predefined nodal stations, rather than indirectly inferring nodal status from primary-tumor features alone.

### Model development

The selected features were subsequently entered into five representative classifiers spanning different paradigms of tabular learning, including decision tree, random forest, light gradient boosting machine (LightGBM), extreme gradient boosting, and tabular prior-data fitted network (TabPFN). TabPFN is a recently developed transformer-based model for tabular data and was included to provide a more contemporary comparator alongside established tree-based and boosting-based methods [21]. Model development was performed in the training cohort. Hyperparameter tuning for candidate classifiers was performed within the training cohort using five-fold cross-validation and grid search. The validation cohort was not used for feature selection or hyperparameter tuning and was used for final model evaluation and exploratory comparison of prespecified radiomics strategies and classifiers. Based on this comparison, the multi-station lymph-node radiomics strategy was retained as the final radiomics strategy, and the corresponding best-performing model was used for subsequent analyses.

### Clinical model, clinical-radiomic model, and nomogram

A clinical model was constructed using preoperative clinical variables, including gender, cancer antigen 125 (CA125), tumor size, and clinical T stage. Univariate logistic regression analysis of the clinical variables listed in Supplementary Table 2 did not identify any statistically significant predictors of LNM. Therefore, rather than relying solely on univariate significance, a subset of preoperative clinical variables was selected for model construction based on prior literature [22, 23], clinical relevance, and model parsimony. Based on the selected final radiomics strategy, the radiomics probability was combined with the selected preoperative clinical variables to build a clinical-radiomic model, from which a clinical-radiomic nomogram was subsequently derived for individualized preoperative risk estimation of LNM.

### Model interpretation, robustness, and reproducibility

To improve model interpretability, Shapley additive explanations (SHAP) analysis was performed for the final lymph-node radiomics model. SHAP summary, feature-importance, and dependence plots were generated to illustrate the contributions and potential interactions of the selected radiomics features. To assess model robustness, five-fold cross-validation was performed in the training cohort for the final radiomics model and the clinical-radiomic model.

To assess interobserver reproducibility, 30 randomly selected cases were independently re-segmented by an observer experienced in CRC imaging analysis who was blinded to the original segmentation and pathological results. Radiomics features extracted from the two sets of segmentations and intraclass correlation coefficients (ICCs) were computed for all shared quantitative radiomics features. An ICC greater than 0.75 was interpreted as indicating good reproducibility. In addition, a conceptual local deployment workflow was outlined to illustrate the potential future implementation of the proposed model in a web-based clinical decision-support setting.

### Statistical analysis

Categorical variables were summarized as counts and percentages. Differences between the training and validation cohorts were examined using the chi-square test or Fisher’s exact test, as appropriate. Discriminative ability was assessed by receiver operating characteristic (ROC) analysis and quantified using the area under the receiver operating characteristic curve (AUC) with 95% confidence intervals (CIs). The 95% CIs for AUCs were estimated using the DeLong method. Differences in AUC between models were compared using DeLong’s test. AUC was considered the primary threshold-independent metric for model discrimination. Threshold-dependent metrics, including accuracy, sensitivity, specificity, positive predictive value, and negative predictive value, were calculated using a fixed probability threshold of 0.5 for descriptive consistency across models. Because this fixed threshold may not be optimal for all models, especially weakly separated models, threshold-dependent results were interpreted descriptively. To assess internal robustness, five-fold cross-validation was performed within the training cohort for the final radiomics model and the clinical-radiomic model. Cross-validation performance was summarized as fold-specific AUCs and mean AUC ± standard deviation. As a sensitivity analysis, bootstrap resampling with 1,000 iterations was performed in the validation cohort to estimate the uncertainty of the AUC for the final radiomics model. Calibration was evaluated using calibration curves, and potential net benefit was assessed by decision curve analysis (DCA). Interobserver agreement was quantified using ICCs. In addition to the overall radiomics feature reproducibility analysis, ICCs were calculated for the final selected radiomics features and model-predicted probabilities. Pearson and Spearman correlation analyses were also performed to assess the rank-order consistency of model-predicted probabilities between repeated segmentations. All tests were two-tailed, and a *p* value < 0.05 was considered statistically significant. All analyses were conducted using Python (version 3.9.13) and R (version 4.5.2).

## Results

### Patient characteristics

Following application of the inclusion and exclusion criteria, 183 patients with pathologically confirmed CRC were included in the study (Fig. 1). The cohort was randomly divided into a training cohort (*n* = 128) and a validation cohort (*n* = 55) at a ratio of 7:3. Baseline clinical characteristics for the two cohorts are summarized in Table 1. No statistically significant differences were found between the cohorts (all *p* > 0.05). Pathological nodal-burden characteristics, including pathological N stage, harvested lymph-node number, and positive lymph-node number, are summarized in Supplementary Table 8.

**Table 1:** Clinical characteristics of patients in the training and validation cohorts

Variable	Training cohort (*n* = 128)	Validation cohort (*n* = 55)	*p* value
Sex			
Female	61 (47.7%)	24 (43.6%)	0.632
Male	67 (52.3%)	31 (56.4%)	
Age (years)			
< 65	48 (37.5%)	25 (45.5%)	0.328
≥ 65	80 (62.5%)	30 (54.5%)	
Body mass index (kg/m²)			
< 24	82 (64.1%)	34 (61.8%)	0.867
≥ 24	46 (35.9%)	21 (38.2%)	
Tumor location			
Colon	69 (53.9%)	36 (65.5%)	0.192
Rectum	59 (46.1%)	19 (34.5%)	
Tumor size (cm)			
< 5	94 (73.4%)	37 (67.3%)	0.475
≥ 5	34 (26.6%)	18 (32.7%)	
Differentiation			
Poor	30 (23.4%)	16 (29.1%)	0.555
Moderate	83 (64.8%)	31 (56.4%)	
Well	15 (11.7%)	8 (14.5%)	
Clinical tumor staging			
T1–2	42 (32.8%)	14 (25.5%)	0.383
T3–4	86 (67.2%)	41 (74.5%)	
CEA			
Negative	116 (90.6%)	52 (94.5%)	0.558
Positive	12 (9.4%)	3 (5.5%)	
CA19-9			
Negative	114 (89.1%)	47 (85.5%)	0.470
Positive	14 (10.9%)	8 (14.5%)	
CA125			
Negative	121 (94.5%)	51 (92.7%)	0.736
Positive	7 (5.5%)	4 (7.3%)	
LNM			
Negative	80 (62.5%)	34 (61.8%)	1
Positive	48 (37.5%)	21 (38.2%)	

Data are presented as number (%). Differences between the training and validation cohorts were compared using the chi-square test or Fisher’s exact test

### Development of the final lymph-node radiomics signature

**Fig. 2:**
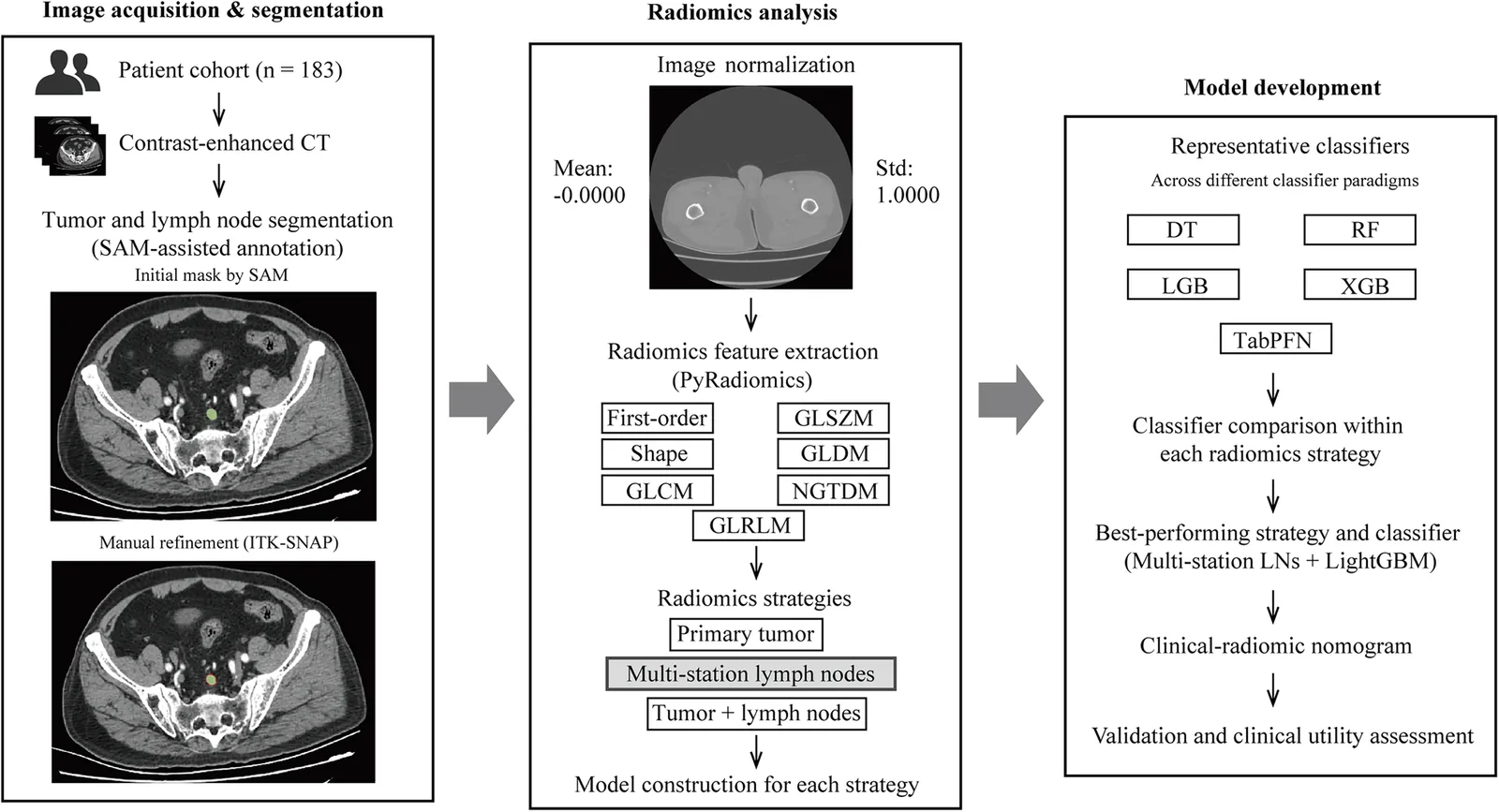
Overall workflow of the study. The workflow included SAM-assisted interactive segmentation of the primary tumor and regional lymph nodes on preoperative contrast-enhanced CT, radiomic feature extraction, development and comparison of three radiomics strategies (primary-tumor, multi-station lymph-node, and combined radiomics), selection of the final radiomics model, integration with preoperative clinical variables, and construction and validation of the final clinical-radiomic nomogram

**Fig. 3:**
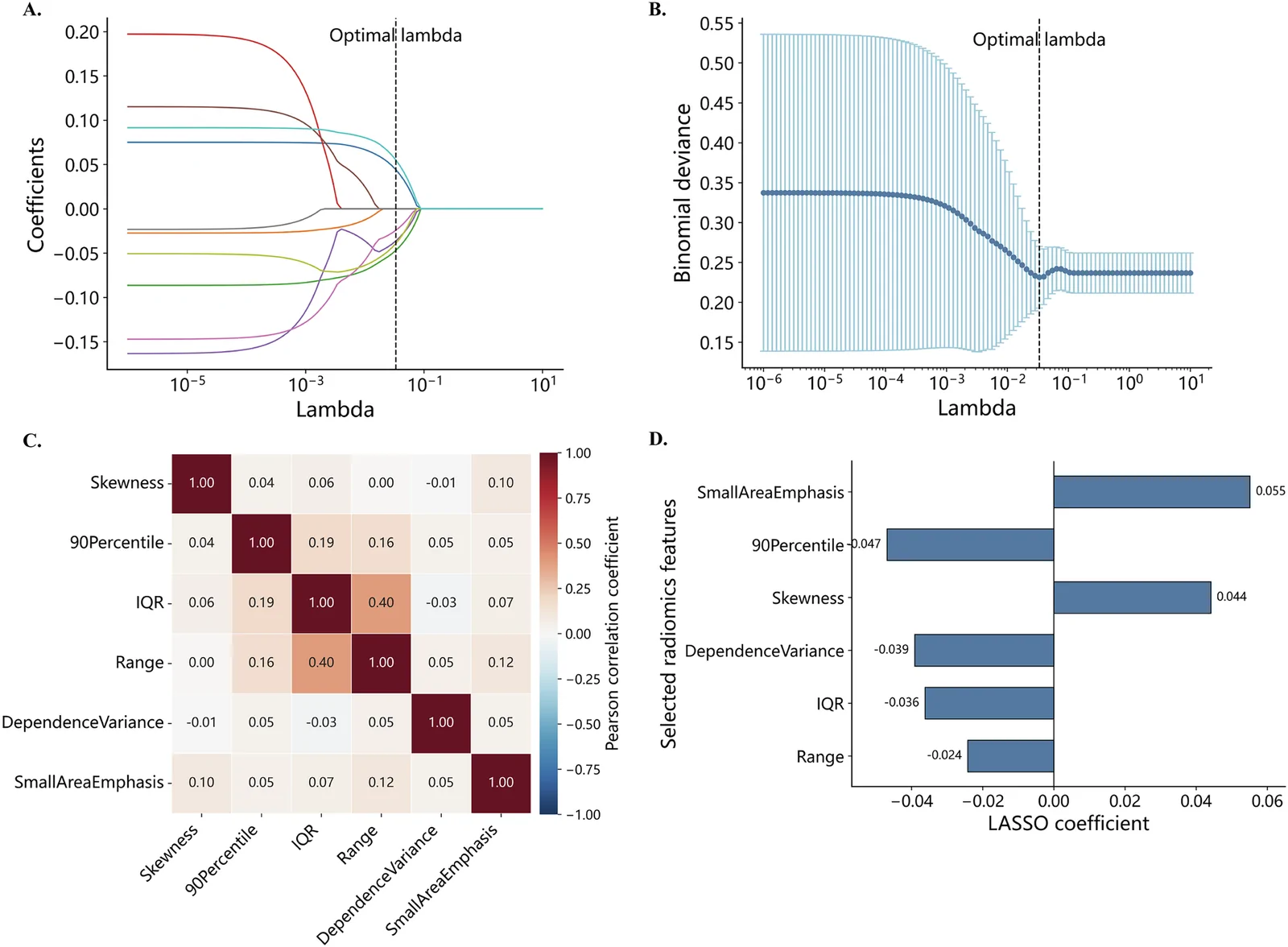
Feature selection and characterization of the final lymph-node radiomics signature. **A** LASSO coefficient trajectories of the candidate radiomic features. **B** Ten-fold cross-validation curve for identifying the optimal regularization parameter in the LASSO model. **C** Correlation heatmap of the selected radiomic features. **D** Relative importance or coefficients of the selected radiomic features incorporated into the final lymph-node radiomics signature

The overall study workflow is shown in Fig. 2. The distribution of segmented lymph-node components across D1, D2, and D3 stations is summarized in Supplementary Table 7. Using the lymph-node radiomics strategy, feature selection was performed with the LASSO method, and the selected features were incorporated into the final lymph-node radiomics signature (Fig. 3). The final lymph-node radiomics model included six selected radiomics features, and their full names, feature classes, image filters, aggregation statistics, and extraction settings are summarized in Supplementary Table 10. Correlation analysis and coefficient visualization further characterized the relationships and relative contributions of the selected lymph-node radiomics features (Fig. 3).

### Comparison of the three radiomics strategies

**Fig. 4:**
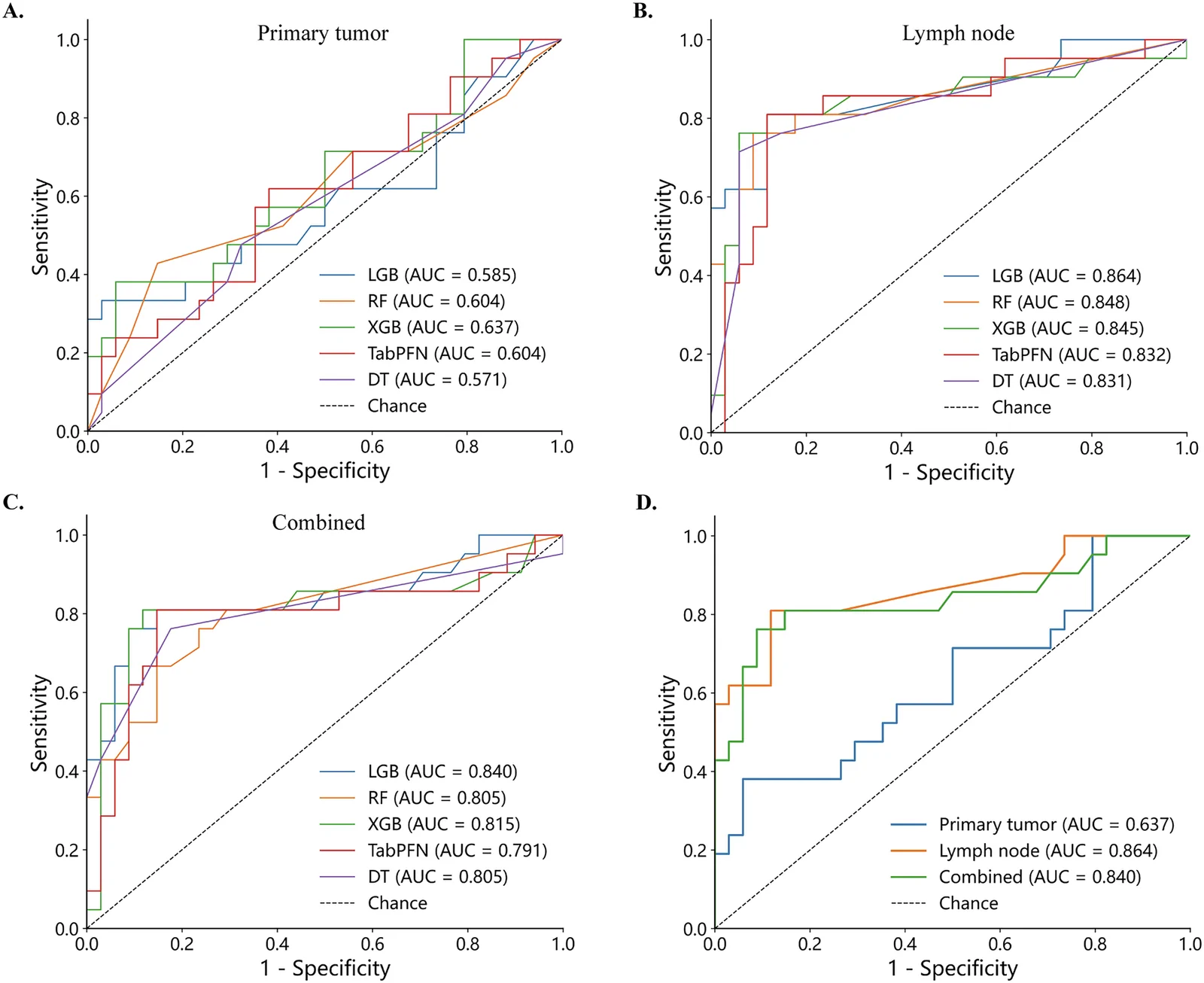
Comparison of the primary-tumor, lymph-node, and combined radiomics strategies in the validation cohort. **A** ROC curves of the five representative classifiers for the primary-tumor radiomics strategy. **B** ROC curves of the five representative classifiers for the lymph-node radiomics strategy. **C** ROC curves of the five representative classifiers for the combined radiomics strategy. **D** ROC curves of the best-performing models from the three radiomics strategies

We first compared three radiomics strategies for preoperative prediction of LNM, including primary-tumor radiomics, lymph-node radiomics, and combined radiomics integrating both feature sets. ROC analyses of the five representative classifiers for each strategy in the validation cohort are shown in Fig. 4, while the corresponding ROC curves in the training cohort are presented in Supplementary Fig. 2. The best-performing primary-tumor radiomics model achieved an AUC of 0.637, whereas the best-performing lymph-node radiomics model, based on the LightGBM classifier, achieved an AUC of 0.864. The best-performing combined radiomics model achieved an AUC of 0.840.

Pairwise comparisons using DeLong’s test showed that the lymph-node radiomics model significantly outperformed the primary-tumor radiomics model (AUC, 0.864 *versus* 0.637; *p*** = **0.0055). The combined radiomics model did not significantly outperform the lymph-node radiomics model (AUC, 0.840 *versus* 0.864; *p*** = **0.2211), but significantly outperformed the primary-tumor radiomics model (AUC, 0.840 *versus* 0.637; *p*** = **0.0210) (Supplementary Table 1). Because the combined radiomics strategy did not provide additional discriminatory benefit over the lymph-node radiomics strategy, the lymph-node radiomics model was retained as the final radiomics model for subsequent analyses.

### Comparison of the clinical model, final radiomics model, and clinical-radiomic model

**Fig. 5:**
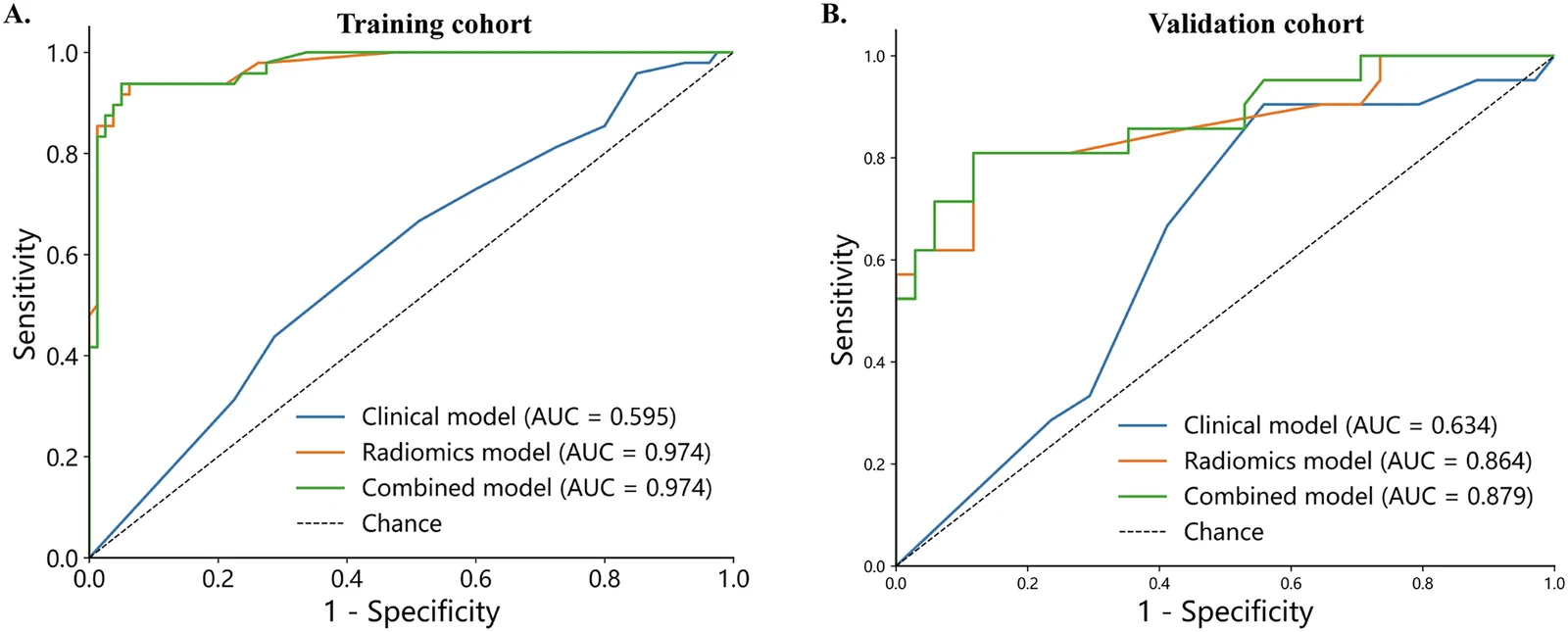
ROC curves of the clinical, final radiomics, and clinical-radiomic models in the training and validation cohorts. **A** ROC curves of the clinical model, final radiomics model, and combined model in the training cohort. **B** ROC curves of the clinical model, final radiomics model, and combined model in the validation cohort

Using the selected lymph-node radiomics strategy, we further evaluated the clinical model, the final radiomics model, and the clinical-radiomic model integrating radiomics with preoperative clinical variables. Their discriminative performances in the training and validation cohorts are summarized in Table 2, and the corresponding ROC curves are presented in Fig. 5. The diagnostic performance of conventional CT-based nodal assessment is summarized in Supplementary Table 9. Conventional CT assessment achieved an accuracy of 69.9%, sensitivity of 72.5%, specificity of 68.4%, and binary cN assessment AUC of 0.704 for patient-level pathological LNM prediction. In the validation cohort, the clinical model yielded an AUC of 0.634, whereas the final radiomics model and the clinical-radiomic model achieved AUCs of 0.864 and 0.879, respectively.

**Table 2:** Discriminative performance of the clinical, radiomics, and clinical-radiomic models in the training and validation cohorts

Cohort	Model	AUC (95% CI)	Accuracy	Sensitivity	Specificity
Training	Clinical	0.595 (0.492–0.693)	0.625	0.000	1.000
Training	Radiomics	0.974 (0.946–0.994)	0.930	0.938	0.925
Training	Clinical-radiomic	0.974 (0.946–0.994)	0.930	0.896	0.950
Validation	Clinical	0.634 (0.480–0.774)	0.618	0.000	1.000
Validation	Radiomics	0.864 (0.746–0.962)	0.836	0.810	0.853
Validation	Clinical-radiomic	0.879 (0.769–0.967)	0.836	0.810	0.853

Data are presented as AUC with 95% CIs, accuracy, sensitivity, and specificity. The radiomics model was constructed using the LightGBM classifier. The clinical-radiomic model incorporated radiomics probability and clinical variables

DeLong’s test indicated that both the final radiomics model and the clinical-radiomic model significantly outperformed the clinical model (*p* = 0.0199 and *p* = 0.0069, respectively). Although the clinical-radiomic model yielded the highest numerical AUC, its difference from the final radiomics model was not statistically significant (*p* = 0.2809) (Supplementary Table 4). Confusion matrices for the final radiomics model and the clinical-radiomic model in the validation cohort are provided in Supplementary Fig. 3. At the fixed probability threshold of 0.5, the clinical model showed limited sensitivity in both cohorts, including a sensitivity of 0 in the validation cohort. This finding suggests that the fixed threshold of 0.5 was not suitable for this weakly separated clinical model. Therefore, threshold-dependent metrics were interpreted descriptively, whereas AUC was used as the primary threshold-independent metric for model comparison. The final radiomics and clinical-radiomic models showed improved threshold-dependent performance compared with the clinical model. In the validation cohort, however, the threshold-dependent metrics of the final radiomics model and clinical-radiomic model were identical.

### Clinical-radiomic nomogram and model evaluation

**Fig. 6:**
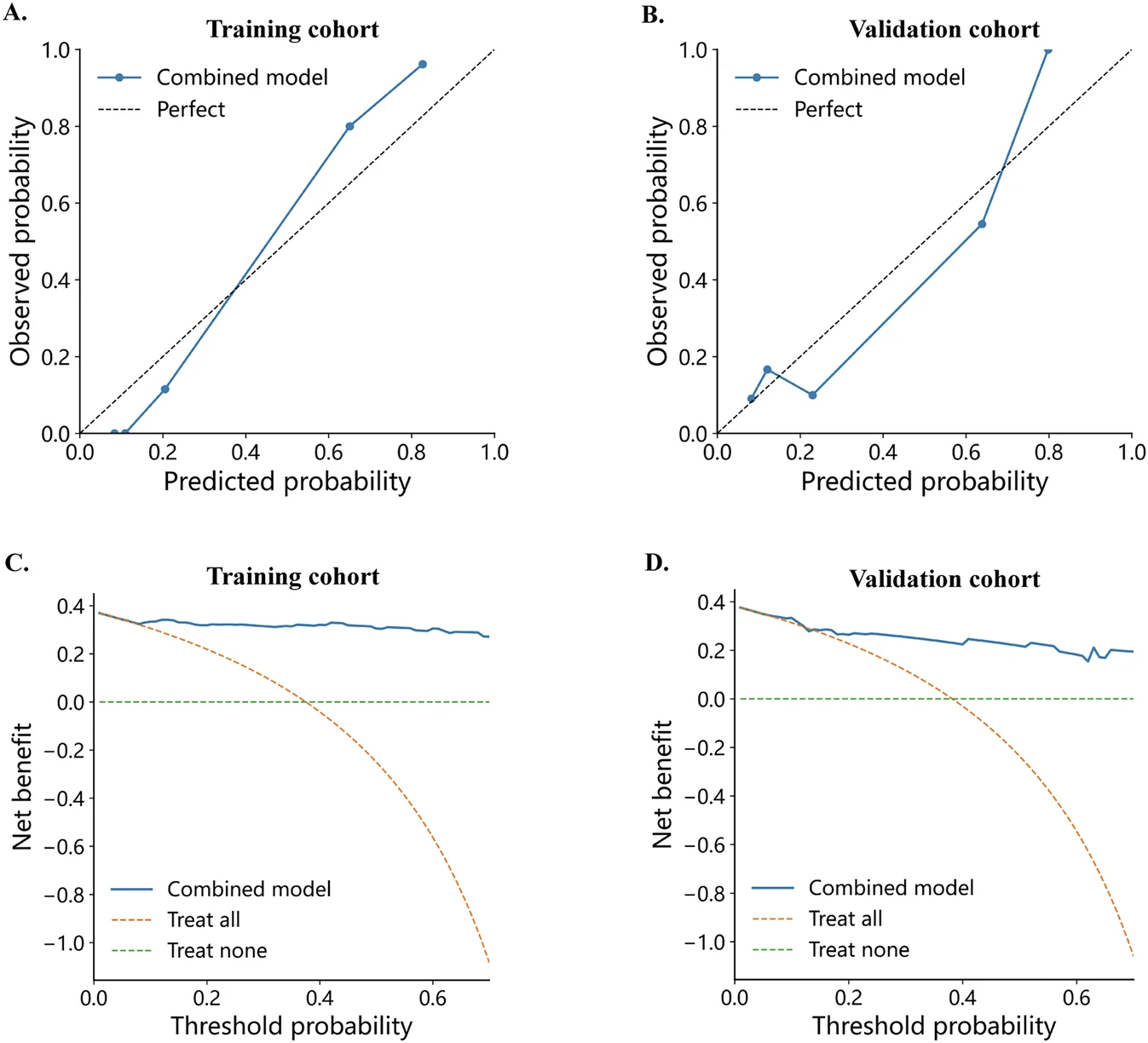
Calibration and decision curve analyses of the clinical-radiomic model in the training and validation cohorts. **A** Calibration curve of the combined model in the training cohort. **B** Calibration curve of the combined model in the validation cohort. The diagonal dashed line represents perfect agreement between predicted and observed probabilities; closer proximity of the calibration curve to this reference line indicates better calibration performance. **C** DCA of the combined model in the training cohort. **D** DCA of the combined model in the validation cohort. The net benefit of the combined model was compared with the treat-all and treat-none strategies across a range of threshold probabilities to evaluate its potential clinical utility

A clinical-radiomic nomogram was subsequently constructed based on the clinical-radiomic model to provide individualized preoperative prediction of LNM (Supplementary Fig. 4). Calibration curves in both the training and validation cohorts showed acceptable agreement between the predicted and observed probabilities, and DCA indicated that the clinical-radiomic model yielded greater net benefit than the treat-all and treat-none strategies across a range of threshold probabilities (Fig. 6). Overall, the nomogram should be interpreted as an interpretable visualization of the integrated clinical-radiomic model for individualized risk estimation, rather than as evidence of a statistically superior model compared with lymph-node radiomics alone.

### Model interpretation, robustness, and reproducibility

To further interpret the final lymph-node radiomics model, SHAP analysis was performed. SHAP summary and importance plots demonstrated the relative contributions of the selected lymph-node radiomics features to the model output (Supplementary Fig. 5). SHAP dependence plots further illustrated the nonlinear effects and potential interactions of the selected radiomics features within the LightGBM model (Supplementary Fig. 6).

Five-fold cross-validation demonstrated good internal robustness of both the final radiomics model and the clinical-radiomic model in the training cohort (Supplementary Fig. 7 and Supplementary Table 3). In the validation-cohort bootstrap sensitivity analysis, the final radiomics model showed a bootstrap mean AUC of 0.865, with a 95% CI of 0.744–0.960 across 1,000 bootstrap iterations (Supplementary Table 6). The relatively wide CI reflects the limited size of the validation cohort. Inter-observer reproducibility analysis showed that the majority of radiomics features had good to excellent agreement, supporting the robustness of the feature extraction procedure (Supplementary Fig. 8 and Supplementary Table 5). Among 2007 extracted features, 1,769 (88.1%) showed excellent reproducibility (ICC ≥ 0.90), and 1,876 (93.5%) showed good or excellent reproducibility (ICC ≥ 0.75). The mean ICC was 0.938. The ICCs of the final selected radiomics features and model-predicted probabilities are summarized in Supplementary Table 11. Five of the six final selected radiomics features showed good-to-excellent interobserver reproducibility, whereas one GLSZM feature showed limited reproducibility. For model-predicted probabilities, the absolute-agreement ICC was limited; however, Pearson and Spearman correlation analyses showed high consistency in prediction ranking between repeated segmentations.

As an exploratory step toward future implementation, we outlined a conceptual local deployment workflow for web-based preoperative prediction of LNM, integrating image input, lymph-node mask upload, clinical variable entry, automated radiomics processing, and model inference (Supplementary Fig. 9).

## Discussion

In this proof-of-concept study, we systematically investigated three CT-based radiomics approaches for preoperative prediction of LNM in CRC, encompassing primary-tumor radiomics, multi-station lymph-node radiomics, and an integrated radiomics strategy. Three main findings emerged. First, the multi-station lymph-node radiomics strategy significantly outperformed the primary-tumor radiomics strategy in the validation cohort. Second, although the combined radiomics strategy improved performance over the primary-tumor strategy, it did not significantly outperform the lymph-node radiomics strategy, which remained the best-performing radiomics approach. Third, the clinical-radiomic model showed acceptable calibration and potential net benefit, but provided only limited incremental discriminative value beyond the final radiomics model. Collectively, these findings suggest that direct multi-station lymph-node characterization may provide complementary imaging information beyond conventional primary-tumor-based radiomics for patient-level preoperative LNM prediction in CRC.

This study moved beyond the conventional primary-tumor-centered radiomics framework. Most previous radiomics studies in CRC have attempted to infer lymph node status indirectly from features extracted from the primary lesion [12, 24–27]. While clinically convenient, this approach may not fully capture the phenotypic heterogeneity of regional lymph nodes, which are more directly related to metastatic dissemination. In contrast, lymph-node-based radiomics studies in CRC remain limited and have mainly focused on mesorectal lymph nodes, lateral pelvic lymph nodes, or selected nodal stations [28–30], mostly in rectal cancer and predominantly using MRI. Tong et al reported an AUC of 0.76 using lymph nodes around the primary tumor [13]. In contrast, our lymph-node radiomics strategy was designed to characterize nodal phenotypes across three predefined nodal stations, thereby providing information that is biologically closer to the actual metastatic process. This may explain why the multi-station lymph-node radiomics model significantly outperformed the primary-tumor model in our cohort. Our findings are also in agreement with prior studies showing that direct lymph-node analysis may improve preoperative prediction of nodal involvement [16], although earlier work generally focused only on the largest regional lymph node.

This lymph-node-centered strategy is clinically relevant because conventional CT assessment may miss metastatic lymph nodes that remain normal in size and morphology. By extracting radiomics features from visible regional lymph nodes across D1, D2, and D3 stations, our approach was intended to capture subvisual nodal information beyond conventional size- or morphology-based assessment. Compared with conventional CT-based nodal assessment, which showed moderate diagnostic performance, the multi-station lymph-node radiomics model provided additional quantitative imaging information for patient-level nodal risk stratification. However, CT-based radiomics remains limited by CT spatial resolution and cannot directly detect microscopic metastases in non-visible lymph nodes. FDG-PET/CT was not routinely performed in our cohort, precluding direct comparison with PET-based nodal assessment. Therefore, the proposed model should be interpreted as a potential adjunct to contrast-enhanced CT for patient-level nodal risk stratification, rather than a replacement for other imaging modalities or pathological evaluation.

Another notable finding is that the combined radiomics strategy, although significantly superior to the primary-tumor radiomics strategy, did not significantly outperform the multi-station lymph-node radiomics strategy. This result is methodologically and biologically informative. It suggests that once regional lymph-node phenotypes are directly characterized across multiple nodal stations, adding primary-tumor radiomics features may contribute only limited information. From a translational perspective, this is advantageous, because a lymph-node-centered radiomics strategy may achieve strong predictive performance while preserving methodological simplicity and clearer biological interpretability. Accordingly, we retained the lymph-node radiomics model as the final radiomics strategy for subsequent analyses. This decision was further supported by the fact that the final radiomics model also significantly outperformed the clinical model, whereas the improvement provided by the combined model over radiomics alone remained modest.

The clinical-radiomic nomogram may provide an interpretable visualization of the integrated model for individualized patient-level preoperative risk estimation. In this study, the clinical-radiomic model demonstrated acceptable calibration and favorable decision-curve performance; however, its incremental value over the final radiomics model alone was limited. The improvement in validation AUC was small and not statistically significant, and the threshold-dependent metrics were identical between the two models in the validation cohort. Therefore, the nomogram should be interpreted primarily as a clinically interpretable risk-visualization tool rather than as evidence of a substantially superior predictive model.

Interpretability and reproducibility are particularly important for radiomics studies intended for translational application. To improve interpretability, we applied SHAP analysis to the final LightGBM-based lymph-node radiomics model and visualized the relative contributions and nonlinear effects of the selected features. In addition, five-fold cross-validation supported the internal robustness of the radiomics and combined models, and inter-observer ICC analysis demonstrated high reproducibility of the extracted radiomics features, with 93.5% of features showing ICC values ≥ 0.75 and 88.1% showing ICC values ≥ 0.90. These results strengthen the methodological reliability of our pipeline. Furthermore, we outlined a conceptual local deployment workflow for future web-based implementation. This workflow was intended as a conceptual demonstration rather than a validated clinical deployment system.

However, several limitations should be acknowledged. First, this was a single-center retrospective study with a moderate sample size, particularly in the validation cohort. Although preprocessing, feature selection, and hyperparameter tuning were restricted to the training cohort, and training-cohort five-fold cross-validation supported internal robustness, multiple radiomics strategies and classifiers were compared in this proof-of-concept study, which may introduce residual model-selection optimism. The validation-cohort bootstrap analysis showed a bootstrap mean AUC consistent with the primary validation AUC; however, the CI remained relatively wide, and bootstrap analysis does not replace independent external validation or fully nested validation. Therefore, the validation performance should be interpreted cautiously, and larger multicenter cohorts with standardized CT acquisition, segmentation protocols, prospective evaluation, and fully nested validation are required before clinical translation.

Second, all included patients underwent surgical resection because postoperative pathological lymph-node status was used as the reference standard, which may have introduced selection bias and limits the applicability of our findings mainly to patients with resectable CRC undergoing preoperative surgical evaluation. In addition, the prediction task was performed at the patient level, and direct node-by-node pathological correlation was not available. Therefore, the model should not be interpreted as diagnosing metastasis in each segmented lymph node. Systematic longitudinal oncologic follow-up was also not included; therefore, associations between the radiomics score and recurrence-free or overall survival could not be evaluated.

Finally, segmentation of small lymph nodes and CT acquisition variability remain potential sources of uncertainty. Although SAM-assisted annotation facilitated initial ROI generation, all masks required manual review and correction, and annotation time was not prospectively recorded; therefore, the current segmentation workflow should be considered research-oriented rather than immediately suitable for routine clinical implementation. Although image-level intensity normalization and image-mask geometric alignment were performed, uniform isotropic voxel resampling and formal scanner harmonization were not applied. Moreover, although the overall extracted radiomics features showed good interobserver reproducibility, one final selected texture feature and the absolute agreement of model-predicted probabilities showed limited reproducibility between repeated segmentations. However, model-predicted probabilities remained highly correlated between repeated segmentations, suggesting that segmentation variability may affect absolute probability values more than relative risk ranking. Future studies should further evaluate automated segmentation, multiphase CT radiomics, scanner harmonization, and model-output robustness.

In conclusion, this single-center proof-of-concept study showed that CT-based multi-station lymph-node radiomics achieved higher validation performance than primary-tumor radiomics for patient-level preoperative prediction of LNM in CRC. Direct characterization of regional lymph nodes may provide additional imaging information for nodal risk stratification. Nevertheless, the proposed model is not ready for direct clinical implementation and requires multicenter external validation, standardized imaging and segmentation protocols, and prospective evaluation before clinical translation.

## Supplementary information

**Additional File 1: Supplementary Fig. 1** Multiplanar and three-dimensional visualization of the primary tumor and regional lymph nodes across three nodal stations. Representative axial, sagittal, coronal, and three-dimensional views show the primary tumor and regional lymph nodes distributed across the D1, D2, and D3 nodal stations. In the three-dimensional schematic, the primary tumor is shown in brown. Regional lymph nodes are color-coded by nodal station as follows: D1, red; D2, blue; and D3, yellow. This illustration demonstrates the segmentation scheme and anatomical distribution of the ROIs used for radiomics analysis. **Supplementary Fig. 2** Supplementary Fig. 2 ROC curves of the five representative classifiers for the primary-tumor, lymph-node, and combined radiomics strategies in the training cohort. (A) Primary-tumor radiomics strategy. (B) Lymph-node radiomics strategy. (C) Combined radiomics strategy. **Supplementary Fig. 3** Confusion matrices of the final radiomics and clinical-radiomic models in the validation cohort. (A) Confusion matrix of the final radiomics model. (B) Confusion matrix of the clinical-radiomic model. The matrices show the numbers of true positives, true negatives, false positives, and false negatives in the validation cohort at the predefined classification threshold. **Supplementary Fig. 4** Clinical-radiomic nomogram for preoperative prediction of lymph node metastasis. The nomogram was constructed using the final lymph-node radiomics signature and selected preoperative clinical variables to provide individualized risk estimation of lymph node metastasis. **Supplementary Fig. 5** SHAP-based interpretation of the final lymph-node radiomics model. (A) SHAP summary plot showing the direction and magnitude of the contribution of each selected radiomic feature to the model output. (B) SHAP feature-importance plot ranking the selected radiomic features according to their mean absolute SHAP values in predicting lymph node metastasis. **Supplementary Fig. 6** SHAP dependence plots of the selected radiomic features in the LightGBM model. (A) Skewness. (B) 90Percentile. (C) IQR. (D) Range. (E) DependenceVariance. (F) SmallAreaEmphasis. Each dot represents one patient. The x-axis shows the feature value, the y-axis shows the corresponding SHAP value, and the color indicates the value of the interacting feature. These plots illustrate the nonlinear effects of individual radiomic features and their potential interactions in the model. **Supplementary Fig. 7** Five-fold cross-validation AUCs of the final radiomics and clinical-radiomic models in the training cohort. The figure shows the distribution of AUCs obtained from five-fold cross-validation for the final radiomics model and the clinical-radiomic model in the training cohort. Each dot represents the AUC of an individual fold. These results reflect the internal robustness and stability of the two models during development. **Supplementary Fig. 8** Distribution of inter-observer ICCs for radiomic features. Histogram of intraclass correlation coefficients (ICCs) for radiomic features extracted from 30 randomly selected cases independently segmented by two observers. The mean ICC value was 0.938, indicating high overall inter-observer reproducibility of the extracted radiomics features. **Supplementary Fig. 9** Conceptual schematic of a proposed local deployment workflow for preoperative prediction of lymph node metastasis. The workflow incorporates contrast-enhanced CT images, lymph-node masks, and preoperative clinical variables, followed by image normalization, mask verification, radiomic feature extraction, feature preprocessing, and model inference to generate the predicted probability of lymph node metastasis and individualized risk stratification. **Supplementary Table 1.** Pairwise comparisons of AUCs among the three radiomics strategies in the validation cohort using DeLong’s test. **Supplementary Table 2.** Univariate logistic regression analysis of preoperative clinical variables in the training cohort. **Supplementary Table 3.** Five-fold cross-validation results of the radiomics and clinical-radiomic models in the training cohort. **Supplementary Table 4.** Pairwise comparisons of AUCs in the validation cohort using DeLong’s test. **Supplementary Table 5.** Summary of inter-observer reproducibility for radiomics features. **Supplementary Table 6.** Validation-cohort bootstrap analysis of model discrimination. **Supplementary Table 7.** Distribution of segmented lymph-node components across D1, D2, and D3 stations. **Supplementary Table 8.** Pathological nodal-burden characteristics of the study cohort. **Supplementary Table 9.** Diagnostic performance of conventional CT-based nodal assessment for pathological lymph-node metastasis. **Supplementary Table 10.** Full list of selected radiomics features in the final lymph-node radiomics model. **Supplementary Table 11.** Reproducibility of final selected radiomics features and model-predicted probabilities.

## Data Availability

The datasets generated and/or analyzed during the current study are not publicly available because they contain potentially identifiable patient information and are subject to institutional data-sharing restrictions. De-identified data may be available from the corresponding author on reasonable request and with permission from the relevant institution.

## Abbreviations

AUC: Area under the receiver operating characteristic curve
CA125: Cancer antigen 125
CI: Confidence interval
CRC: Colorectal cancer
CT: Computed tomography
DCA: Decision curve analysis
ICC: Intraclass correlation coefficient
LASSO: Least absolute shrinkage and selection operator
LightGBM: Light gradient boosting machine
LNM: Lymph node metastasis
ROC: Receiver operating characteristic
ROI: Region of interest
SAM: Segment anything model
SHAP: Shapley additive explanations
