# Supplementary material for: CT-based multi-station lymph-node radiomics for preoperative prediction of lymph node metastasis in colorectal cancer: a proof-of-concept comparison with primary-tumor radiomics
Source: Eur Radiol Exp. 2026 Oct 12;10:153. doi: 10.1186/s41747-026-00814-y (PMC13658740; doi:10.1186/s41747-026-00814-y)
Supplement: Supplementary file 1 [file 41747_2026_814_MOESM1_ESM.pdf]

# CT-based multi-station lymph-node radiomics for preoperative prediction of lymph node metastasis in colorectal cancer: a proof-of-concept comparison with primary-tumor radiomics

**Electronic Supplementary Material**

**Supplementary Figures & Tables**

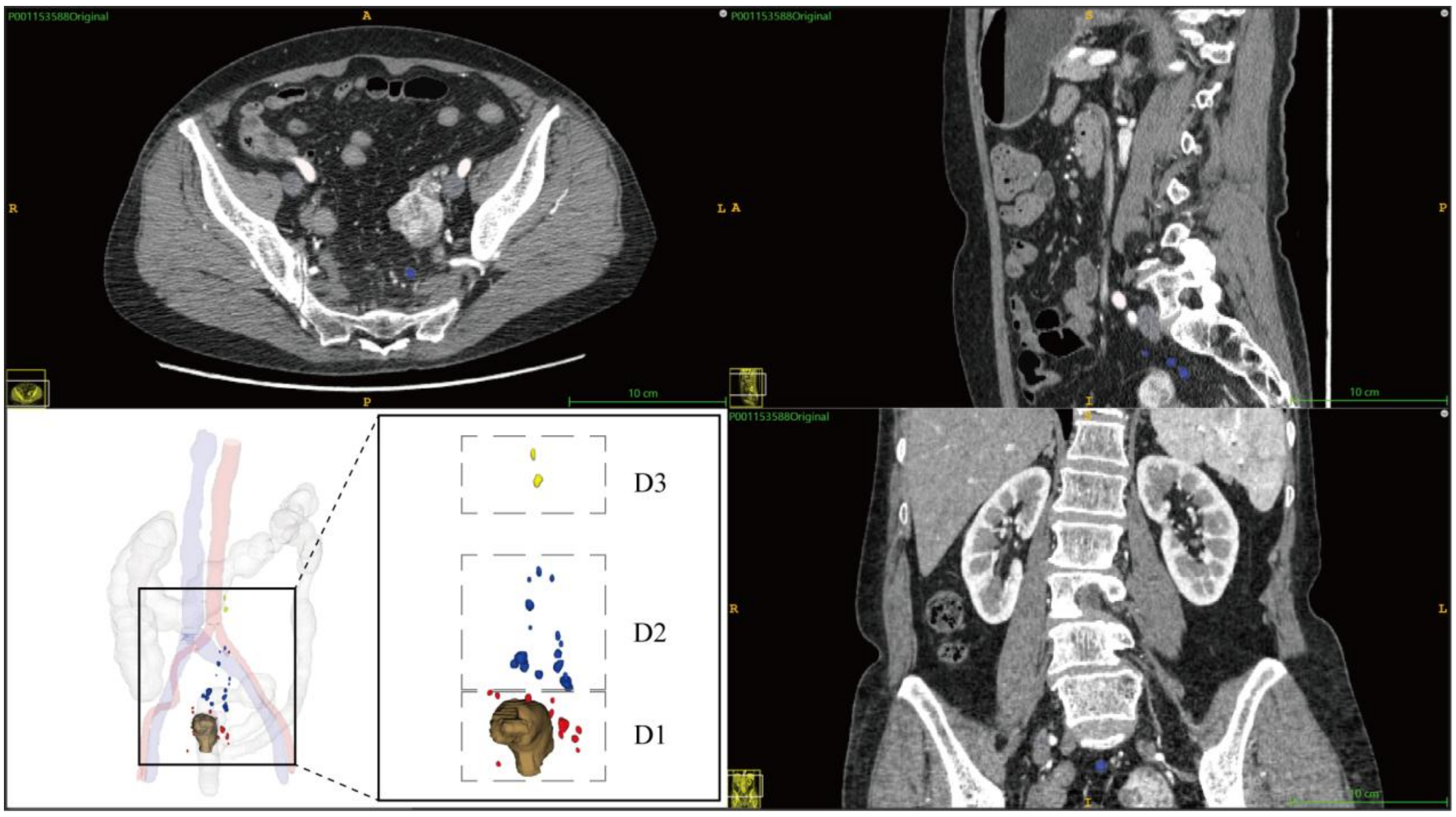


**Supplementary Fig. 1 Multiplanar and three-dimensional visualization of the primary tumor and regional lymph nodes across three nodal stations.** Representative axial, sagittal, coronal, and three-dimensional views show the primary tumor and regional lymph nodes distributed across the D1, D2, and D3 nodal stations. In the three-dimensional schematic, the primary tumor is shown in brown. Regional lymph nodes are color-coded by nodal station as follows: D1, red; D2, blue; and D3, yellow. This illustration demonstrates the segmentation scheme and anatomical distribution of the ROIs used for radiomics analysis.

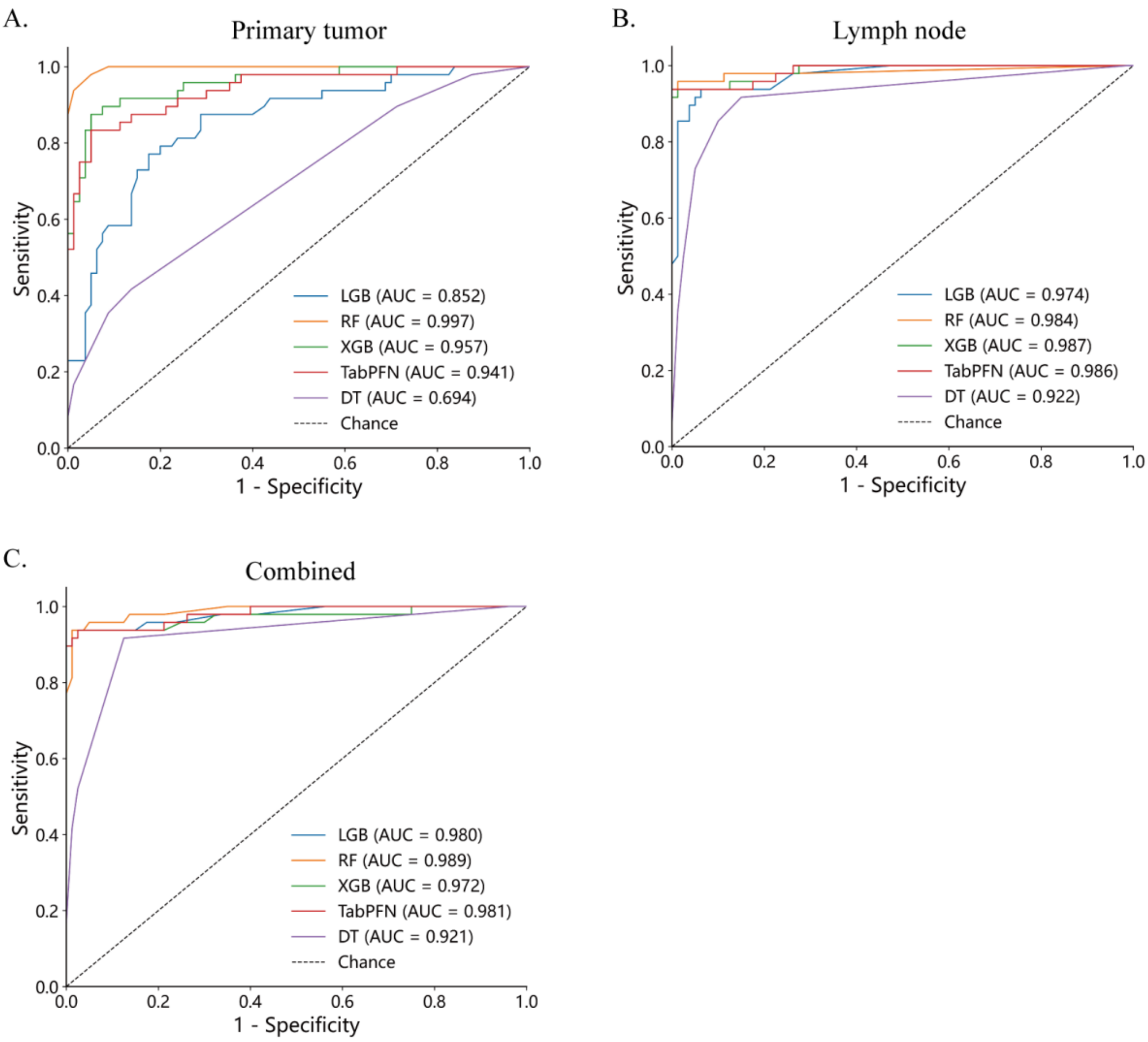


**Supplementary Fig. 2 ROC curves of the five representative classifiers for the primary-tumor, lymph-node, and combined radiomics strategies in the training cohort.**

(A) Primary-tumor radiomics strategy.

(B) Lymph-node radiomics strategy.

(C) Combined radiomics strategy.

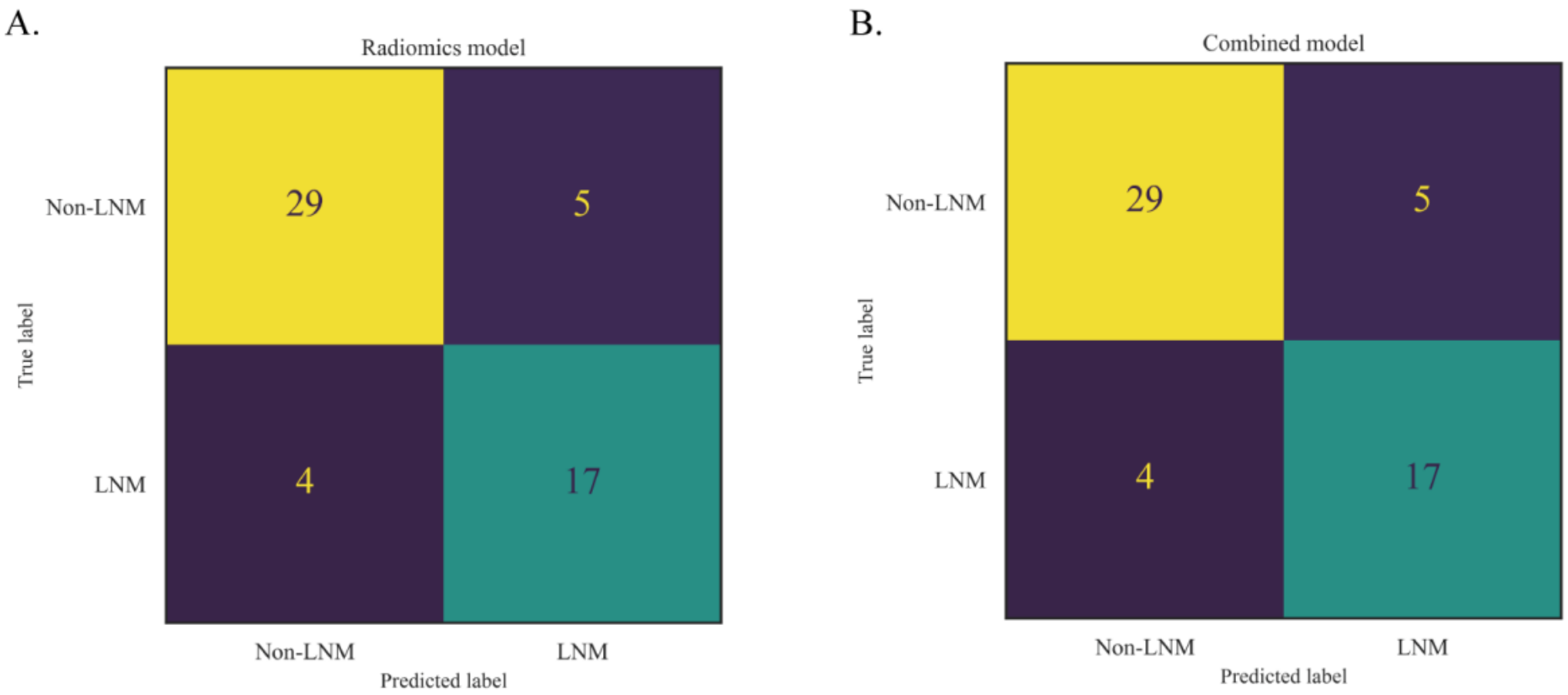


**Supplementary Fig. 3 Confusion matrices of the final radiomics and clinical-radiomic models in the validation cohort.**

(A) Confusion matrix of the final radiomics model.

(B) Confusion matrix of the clinical-radiomic model.

The matrices show the numbers of true positives, true negatives, false positives, and false negatives in the validation cohort at the predefined classification threshold.

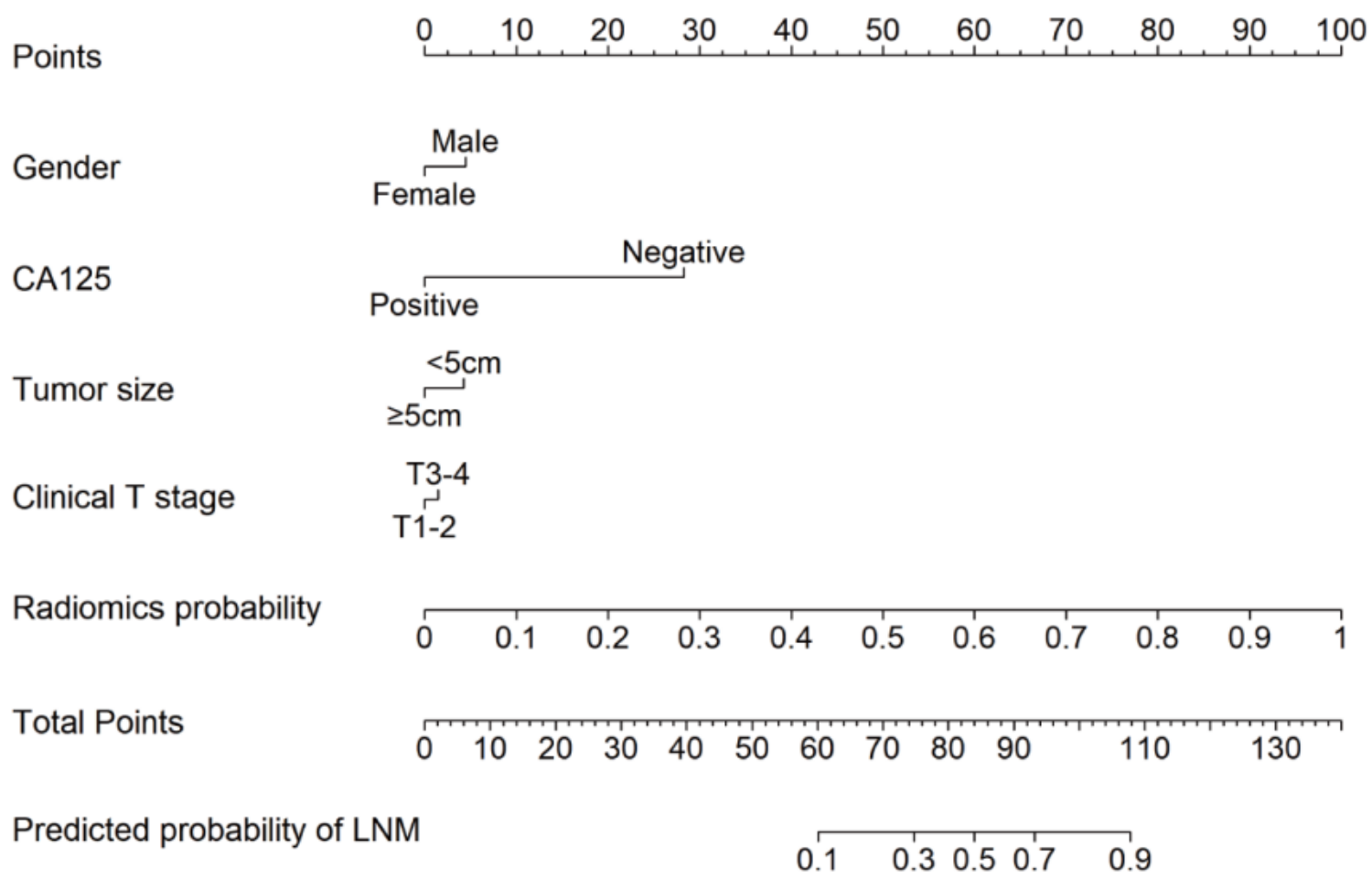


**Supplementary Fig. 4 Clinical-radiomic nomogram for preoperative prediction of lymph node metastasis.** The nomogram was constructed using the final lymph-node radiomics signature and selected preoperative clinical variables to provide individualized risk estimation of lymph node metastasis.

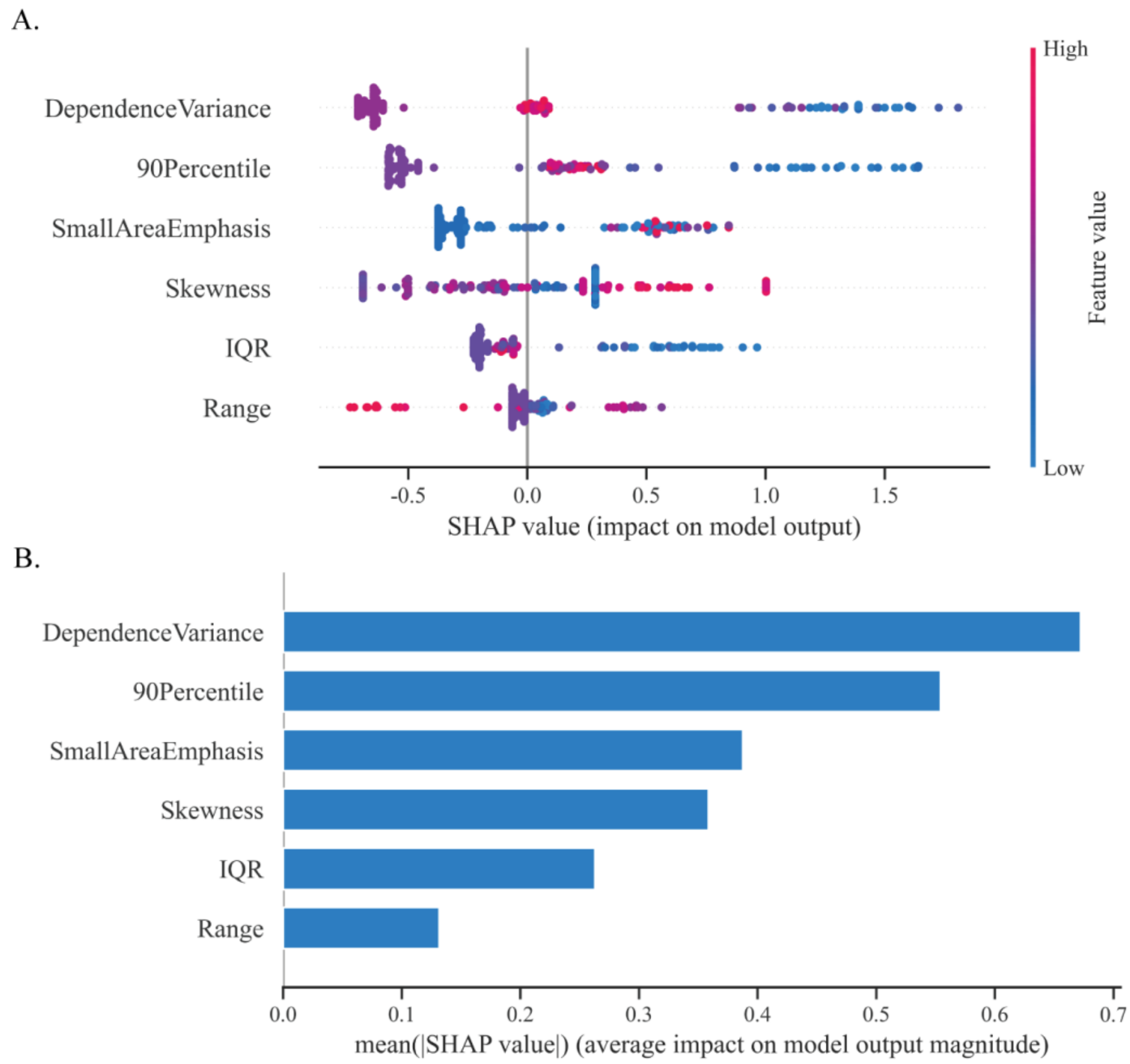


**Supplementary Fig. 5 SHAP-based interpretation of the final lymph-node radiomics model.**

(A) SHAP summary plot showing the direction and magnitude of the contribution of each selected radiomic feature to the model output.

(B) SHAP feature-importance plot ranking the selected radiomic features according to their mean absolute SHAP values in predicting lymph node metastasis.

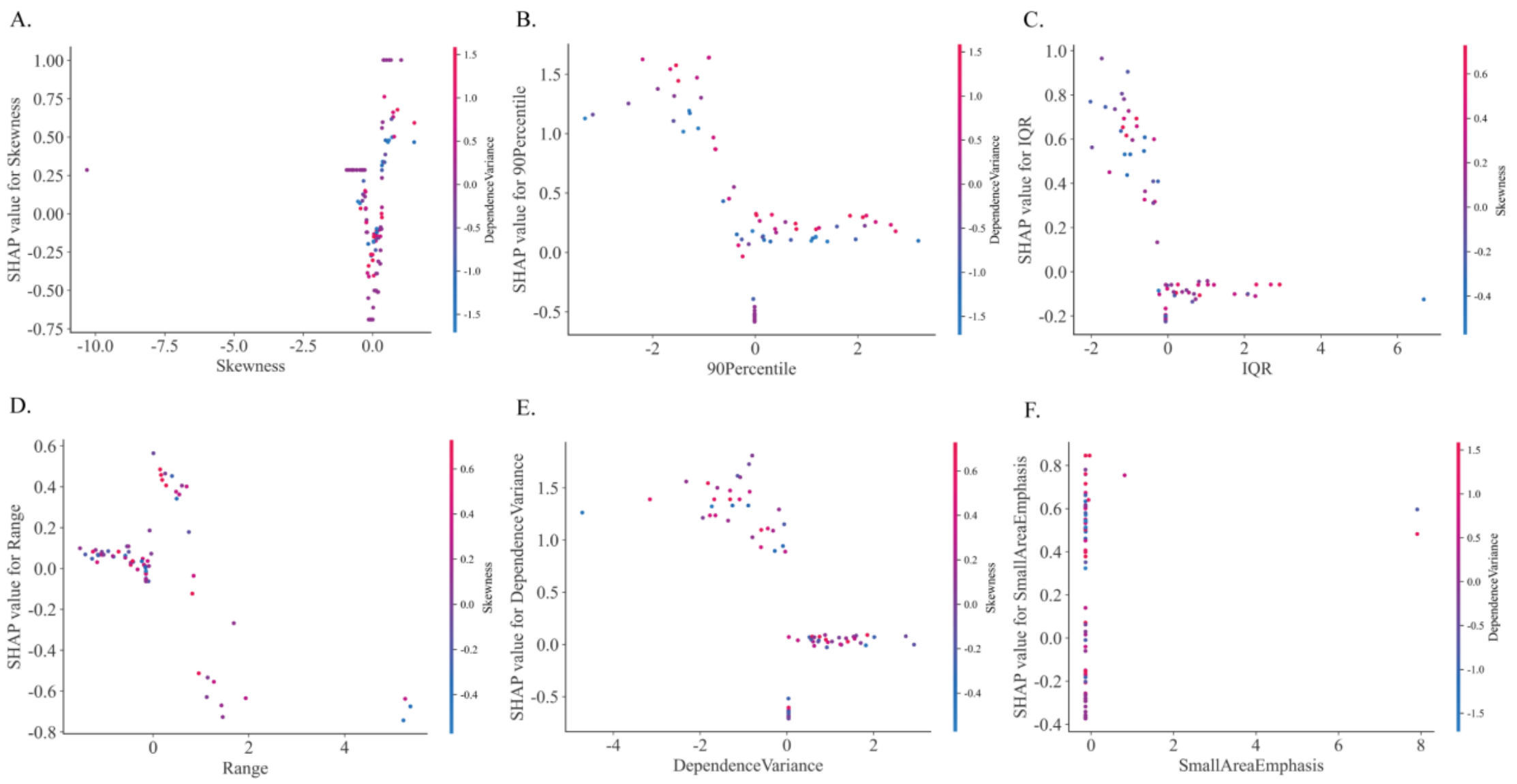


**Supplementary Fig. 6 SHAP dependence plots of the selected radiomic features in the LightGBM model.**

(A) Skewness.

(B) 90Percentile.

(C) IQR.

(D) Range.

(E) DependenceVariance.

(F) SmallAreaEmphasis.

Each dot represents one patient. The x-axis shows the feature value, the y-axis shows the corresponding SHAP value, and the color indicates the value of the interacting feature. These plots illustrate the nonlinear effects of individual radiomic features and their potential interactions in the model.

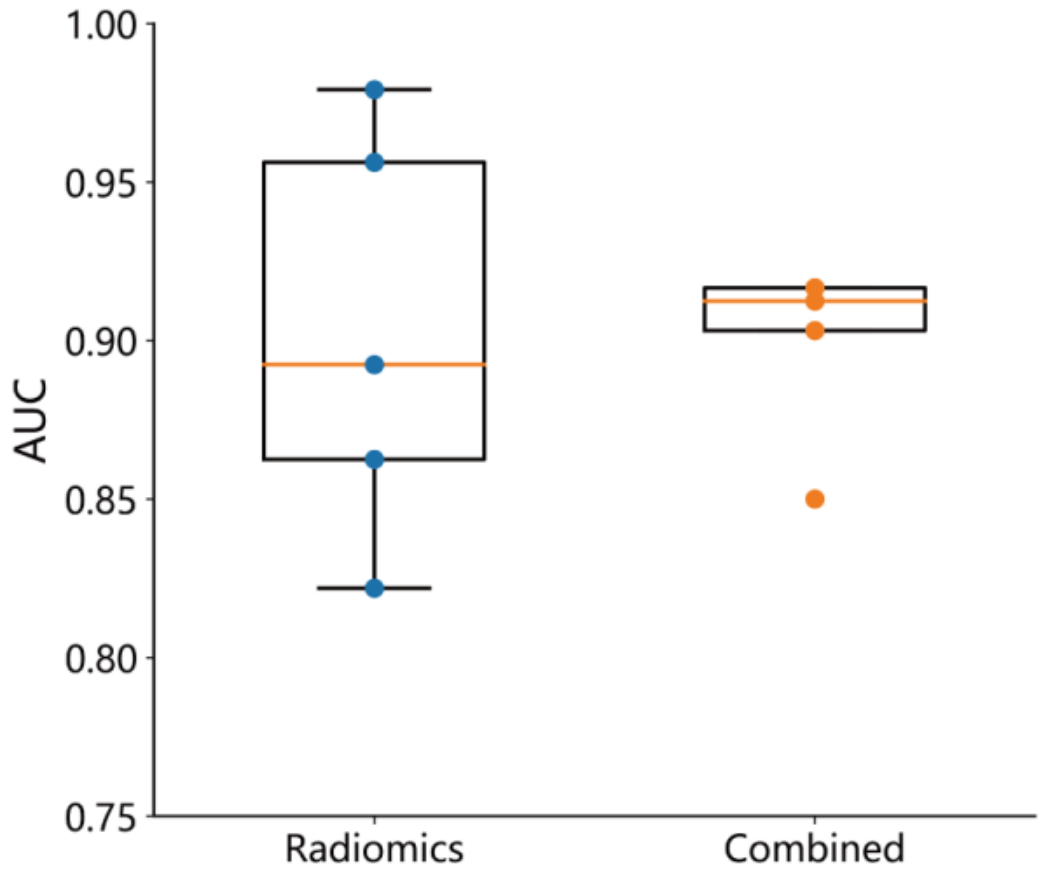


**Supplementary Fig. 7 Five-fold cross-validation AUCs of the final radiomics and clinical-radiomic models in the training cohort.** The figure shows the distribution of AUCs obtained from five-fold cross-validation for the final radiomics model and the clinical-radiomic model in the training cohort. Each dot represents the AUC of an individual fold. These results reflect the internal robustness and stability of the two models during development.

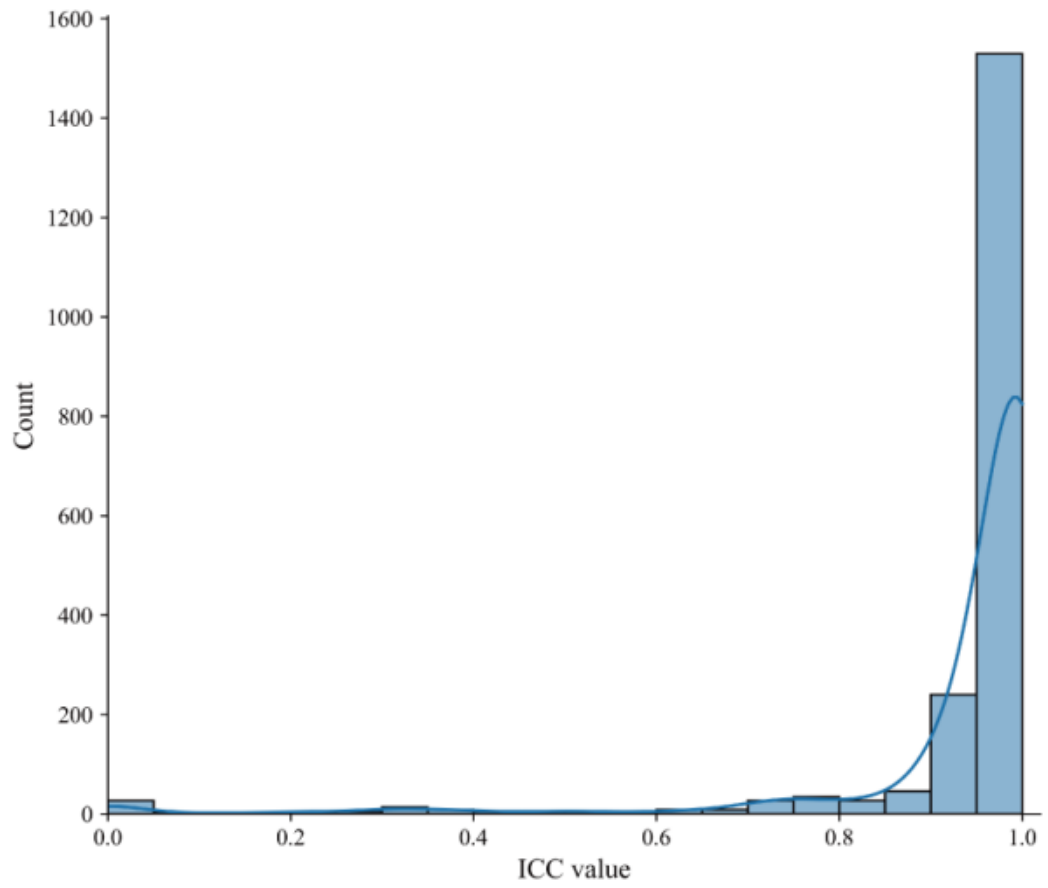


**Supplementary Fig. 8 Distribution of inter-observer ICCs for radiomic features.** Histogram of intraclass correlation coefficients (ICCs) for radiomic features extracted from 30 randomly selected cases independently segmented by two observers. The mean ICC value was 0.938, indicating high overall inter-observer reproducibility of the extracted radiomics features.

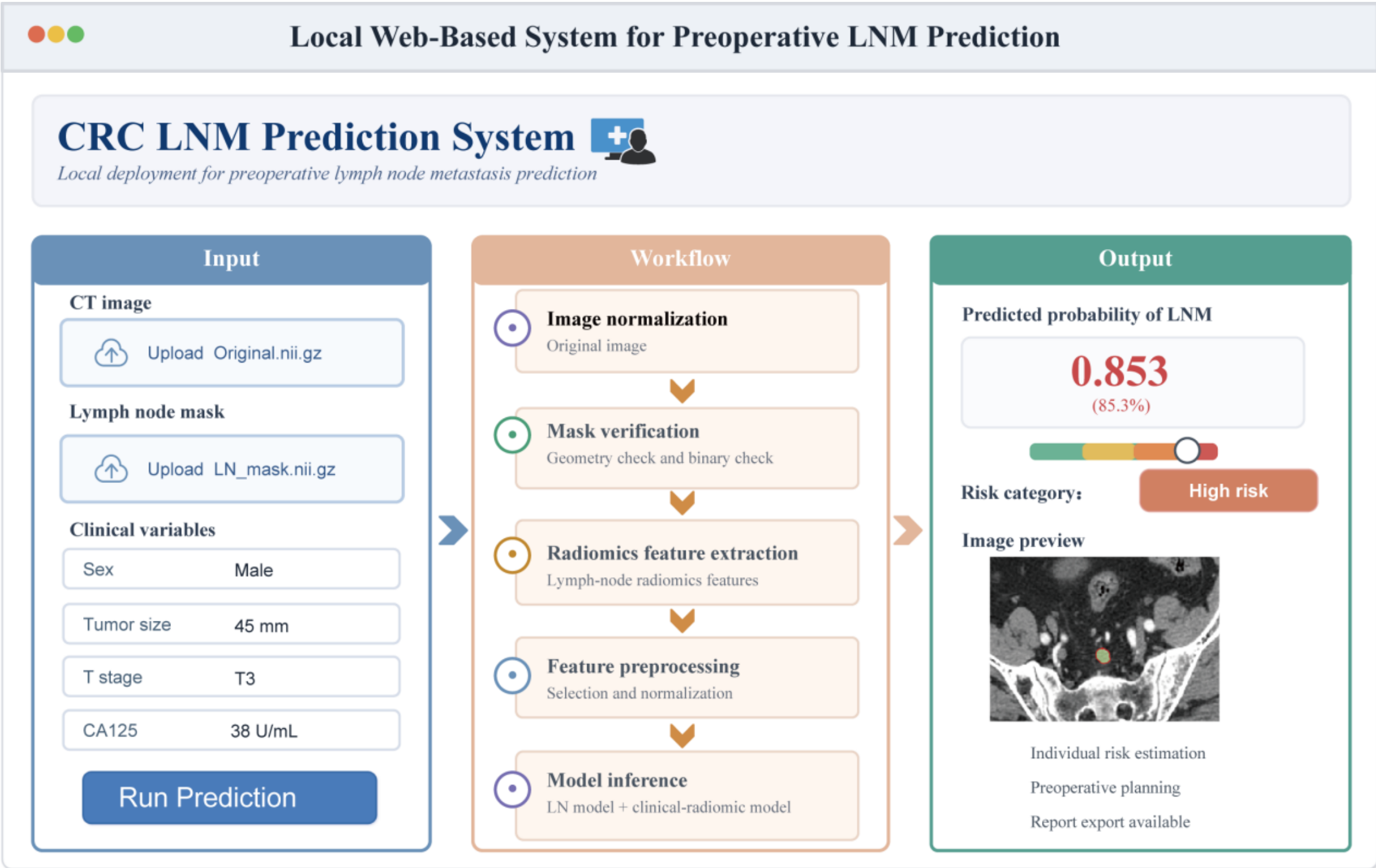


**Supplementary Fig. 9 Conceptual schematic of a proposed local deployment workflow for preoperative prediction of lymph node metastasis.** The workflow incorporates contrast-enhanced CT images, lymph-node masks, and preoperative clinical variables, followed by image normalization, mask verification, radiomic feature extraction, feature preprocessing, and model inference to generate the predicted probability of lymph node metastasis and individualized risk stratification.

**Supplementary Table 1. Pairwise comparisons of AUCs among the three radiomics strategies in the validation cohort using DeLong's test.**

| Comparison | AUC 1 | AUC 2 | Z statistic | P value |
|---|---|---|---|---|
| Primary-tumor vs Lymph-node | 0.637 | 0.864 | 2.778 | 0.0055 |
| Lymph-node vs Combined | 0.864 | 0.840 | 1.223 | 0.2211 |
| Primary-tumor vs Combined | 0.637 | 0.840 | 2.309 | 0.0210 |

Pairwise comparisons of AUCs among the best-performing primary-tumor, lymph-node, and combined radiomics models were performed using DeLong's test in the validation cohort.

**Supplementary Table 2. Univariate logistic regression analysis of preoperative clinical variables in the training cohort.**

| Variables | β | S.E | Z | P | OR (95%CI) |
|---|---|---|---|---|---|
| Gender | | | | | |
| Female | | | | | 1.00 (Reference) |
| Male | 0.25 | 0.37 | 0.68 | 0.493 | 1.29 (0.63 ~ 2.64) |
| Age(years) | | | | | |
| ＜65 | | | | | 1.00 (Reference) |
| ≥65 | -0.42 | 0.37 | -1.13 | 0.259 | 0.65 (0.31 ~ 1.37) |
| Body mass index (kg/m²) | | | | | |
| <24 | | | | | 1.00 (Reference) |
| ≥24 | 0.11 | 0.38 | 0.29 | 0.775 | 1.11 (0.53 ~ 2.34) |
| Tumor location | | | | | |
| Colon | | | | | 1.00 (Reference) |
| Rectum | 0.39 | 0.37 | 1.05 | 0.293 | 1.47 (0.72 ~ 3.02) |
| Tumor size(cm) | | | | | |
| <5 | | | | | 1.00 (Reference) |
| ≥5 | -0.31 | 0.42 | -0.72 | 0.470 | 0.74 (0.32 ~ 1.69) |
| Clinical tumor staging | | | | | |
| T1-2 | | | | | 1.00 (Reference) |
| T3-4 | 0.27 | 0.39 | 0.68 | 0.497 | 1.31 (0.60 ~ 2.83) |
| CEA | | | | | |
| Negative | | | | | 1.00 (Reference) |
| Positive | -0.64 | 0.69 | -0.93 | 0.354 | 0.53 (0.14 ~ 2.05) |
| CA19-9 | | | | | |
| Negative | | | | | 1.00 (Reference) |
| Positive | -0.09 | 0.59 | -0.15 | 0.884 | 0.92 (0.29 ~ 2.92) |
| CA125 | | | | | |
| Negative | | | | | 1.00 (Reference) |
| Positive | -1.34 | 1.10 | -1.22 | 0.222 | 0.26 (0.03 ~ 2.25) |
| Differentiation | | | | | |
| Poor | | | | | 1.00 (Reference) |
| Moderate | -0.44 | 0.43 | -1.01 | 0.313 | 0.65 (0.28 ~ 1.51) |
| Well | -0.88 | 0.69 | -1.27 | 0.203 | 0.42 (0.11 ~ 1.60) |

OR: Odds Ratio, CI: Confidence Interval

**Supplementary Table 3. Five-fold cross-validation results of the radiomics and clinical-radiomic models in the training cohort.**

| Model | Fold 1 AUC | Fold 2 AUC | Fold 3 AUC | Fold 4 AUC | Fold 5 AUC | Mean AUC | SD |
|---|---|---|---|---|---|---|---|
| Radiomics | 0.956 | 0.822 | 0.863 | 0.979 | 0.892 | 0.902 | 0.058 |
| Clinical-radiomic | 0.912 | 0.850 | 0.903 | 0.917 | 0.917 | 0.900 | 0.025 |

AUC, area under the receiver operating characteristic curve; SD, standard deviation. Cross-validation was performed using a 5-fold strategy within the training cohort.

**Supplementary Table 4. Pairwise comparisons of AUCs in the validation cohort using DeLong's test.**

| Comparison | AUC 1 | AUC 2 | Z statistic | *P* value |
|---|---|---|---|---|
| Radiomics vs Clinical-radiomic | 0.864 | 0.879 | 1.078 | 0.2809 |
| Clinical vs Radiomics | 0.634 | 0.864 | 2.327 | 0.0199 |
| Clinical vs Clinical-radiomic | 0.634 | 0.879 | 2.701 | 0.0069 |

AUCs of the clinical, radiomics, and clinical-radiomic models in the validation cohort were compared using DeLong's test.

**Supplementary Table 5. Summary of inter-observer reproducibility for radiomics features.**

| Metric | Value |
|---|---|
| Total features | 2007 |
| Excellent (ICC >= 0.90) | 1769 |
| Good (0.75 <= ICC < 0.90) | 107 |
| Moderate (0.50 <= ICC < 0.75) | 53 |
| Poor (ICC < 0.50) | 78 |
| Median ICC | 0.997435778 |
| Mean ICC | 0.938203535 |

Features were categorized as excellent (ICC ≥ 0.90), good (0.75 ≤ ICC < 0.90), moderate (0.50 ≤ ICC < 0.75), or poor (ICC < 0.50).

**Supplementary Table 6. Validation-cohort bootstrap analysis of model discrimination.**

| Model | Validation AUC | Bootstrap mean AUC | Bootstrap 95% CI | Valid bootstrap iterations |
|---|---|---|---|---|
| Final radiomics model | 0.864 | 0.865 | 0.744-0.960 | 1000 |

AUC, area under the receiver operating characteristic curve. Bootstrap resampling was performed in the validation cohort with 1000 iterations.

**Supplementary Table 7. Distribution of segmented lymph-node components across D1, D2, and D3 stations.**

| Variable | Overall | LNM-negative | LNM-positive |
|---|---|---|---|
| Patients, n | 183 | 114 | 69 |
| Segmented lymph nodes, n | 2702 | 1584 | 1118 |
| Segmented lymph nodes per patient, median (IQR) | 14 (10-18) | 14 (9-17) | 16 (12-19) |
| D1 segmented lymph-node components, n (%) | 1025(37.9) | 561 (35.4) | 464 (41.5) |
| D2 segmented lymph-node components, n (%) | 1314(48.6) | 814 (51.4) | 500 (44.7) |
| D3 segmented lymph-node components, n (%) | 363 (13.4) | 209 (13.2) | 154 (13.8) |
| Patients with D1 lymph-node segmentation, n (%) | 175 (95.6) | 106 (93.0) | 69 (100.0) |
| Patients with D2 lymph-node segmentation, n (%) | 178 (97.3) | 110 (96.5) | 68 (98.6) |
| Patients with D3 lymph-node segmentation, n (%) | 142 (77.6) | 82 (71.9) | 60 (87.0) |

Segmented lymph-node counts were estimated from three-dimensional connected components within station-specific lymph-node masks. Individual node-level short-axis diameters were not retrospectively recorded; therefore, size measurements are not reported.

**Supplementary Table 8. Pathological nodal-burden characteristics of the study cohort.**

| | | | |
|---|---|---|---|
| Patients, n | 183 | 114 | 69 |
| Pathological N stage, n (%) | | | |
| pN0 | 114 (62.3) | 114 (100.0) | 0 (0.0) |
| pN1 | 45 (24.6) | 0 (0.0) | 45 (65.2) |
| pN2 | 24 (13.1) | 0 (0.0) | 24 (34.8) |
| Harvested lymph nodes, total n | 2877 | 1813 | 1064 |
| Harvested lymph nodes per patient, median (IQR) | 15 (12-18) | 15 (12-19) | 15 (12-18) |
| Positive lymph nodes, total n | 250 | 0 | 250 |
| Positive lymph nodes per patient, median (IQR) | 0 (0-2) | 0 (0-0) | 2 (1-5) |
| Patients with ≥12 harvested lymph nodes, n (%) | 154 (84.2) | 94 (82.5) | 60 (87.0) |

Pathological N stage was defined according to the number of metastatic lymph nodes: pN0, no positive lymph nodes; pN1, 1-3 positive lymph nodes; and pN2, ≥4 positive lymph nodes.

**Supplementary Table 9. Diagnostic performance of conventional CT-based nodal assessment for pathological lymph-node metastasis.**

| | |
|---|---|
| Patients, n | 183 |
| Conventional CT-positive, n | 86 |
| Conventional CT-negative, n | 97 |
| True positive, n | 50 |
| False positive, n | 36 |
| True negative, n | 78 |
| False negative, n | 19 |
| Accuracy | 69.9% |
| Sensitivity | 72.5% |
| Specificity | 68.4% |
| Positive predictive value | 58.1% |
| Negative predictive value | 80.4% |
| Binary cN assessment AUC | 0.704 |

For binary cN assessment, cN0 was considered CT-negative and cN1-2 was considered CT-positive. The reported AUC corresponds to the discrimination of this binary assessment.

**Supplementary Table 10. Full list of selected radiomics features in the final lymph-node radiomics model.**

| Selected radiomics feature | Image filter | Feature class | Feature name | Aggregation statistic |
|---|---|---|---|---|
| Original_firstorder_ Skewness__mean__D1 | Original | First-order | Skewness | Mean |
| ALL_original_firstorder_ 90Percentile__mean | Original | First-order | 90th percentile | Mean |
| ALL_original_firstorder_ InterquartileRange__max | Original | First-order | Interquartile range | Maximum |
| ALL_original_firstorder_ Range__mean | Original | First-order | Range | Mean |
| ALL_original_gldm_ DependenceVariance__max | Original | GLDM | Dependence variance | Maximum |
| ALL_original_glszm_ SmallAreaEmphasis__mean | Original | GLSZM | Small area emphasis | Mean |

GLDM, gray-level dependence matrix; GLSZM, gray-level size-zone matrix. All selected features were extracted using PyRadiomics version 3.1.0. Image-level intensity normalization was performed before feature extraction, and no intensity clipping was applied. When image-mask geometric mismatch was detected, masks were resampled to the image space using nearest-neighbor interpolation. Uniform isotropic voxel resampling was not performed. Gray-level discretization was performed using a fixed bin width of 25 gray-level units. The prefix "ALL" indicates pooled lymph-node features aggregated across nodal stations at the patient level.

**Supplementary Table 11. Reproducibility of final selected radiomics features and model-predicted probabilities.**

| Variable | ICC | 95% CI |
|---|---|---|
| Original_firstorder_Skewness__mean__D1 | 0.888 | 0.708-0.996 |
| ALL_original_firstorder_90Percentile__mean | 0.995 | 0.980-0.998 |
| ALL_original_firstorder_InterquartileRange__max | 0.977 | 0.890-0.991 |
| ALL_original_firstorder_Range__mean | 0.925 | 0.612-0.971 |
| ALL_original_gldm_DependenceVariance__max | 0.933 | 0.568-0.973 |
| ALL_original_glszm_SmallAreaEmphasis__mean | 0.318 | 0.000-1.000 |
| Final radiomics model predicted probability | 0.391 | 0.185-0.544 |

| Model output | Pearson r | Spearman ρ |
|---|---|---|
| Final radiomics model predicted probability | 0.852 | 0.911 |

ICC, intraclass correlation coefficient; The predicted probability was generated by applying the trained final lymph-node radiomics model to features extracted from repeated segmentations. Pearson and Spearman correlations were additionally reported to assess rank-order consistency of model-predicted probabilities between repeated segmentations.
